# Nutrient Composition of Marine Fish Species From the East African Coast: Implications for Food and Nutrition Security

**DOI:** 10.1002/fsn3.71159

**Published:** 2026-01-13

**Authors:** Mackrina Patrick Nombo, Betina Lukwambe, Maria Wik Markhus, Talhiya Maulid Ali, Edel O. Elvevoll, Quang Tri Ho, José Mateus Vilanculo, Marian Kjellevold

**Affiliations:** ^1^ School of Aquatic Sciences and Fisheries Technology University of Dar es Salaam Dar es Salaam Tanzania; ^2^ Fisheries Education and Training Agency Bagamoyo Tanzania; ^3^ Institute of Marine Research Bergen Norway; ^4^ School of Natural Science The State University of Zanzibar Zanzibar Tanzania; ^5^ Norwegian College of Fishery Science UiT‐The Arctic University of Norway Tromsø Norway; ^6^ Oceonographic Institute of Mozambique Maputo Mozambique

**Keywords:** aquatic food, EAF‐Nansen Programme, food composition, Mozambique, nutrient reference value, Tanzania

## Abstract

Fish play a significant role in food and nutrition security along the coast of East Africa. However, there is a lack of comprehensive nutrient composition data. This study aimed to present the nutrient composition of the most commonly consumed marine fish species and assess their potential contribution to the Codex nutrient reference values (NRVs) for healthy adults. In total, 123 pooled samples (composites) of 24 commonly consumed marine fish species were collected using pelagic and demersal trawls by the R/V Dr. Fridtjof Nansen during ecosystem surveys along the East African coast in 2018 and 2023. Species were categorized, according to length, into small (< 25 cm) or large (> 25 cm) fish and prepared based on local consumption practices (whole, dressed, headed and gutted, and fillets). All samples were analyzed for macronutrients (protein, fat and fatty acids) and micronutrients (calcium, iron, iodine, zinc, vitamin A, folic acid and vitamin B12). The results show that whole small fish species contain higher levels of various micronutrients, such as calcium, iron, iodine, zinc, vitamin A, and the fatty acids eicosapentaenoic acid (EPA) and docosahexaenoic acid (DHA), compared to the fillets of large fish species. This study showed that several small fish species consumed whole contribute to ≥ 15% of the NRVs of healthy adults for several essential nutrients when a 100 g portion is consumed. The data presented in this study provide a valuable addition to the Tanzanian and Mozambican food composition tables, enhancing the understanding of fish as a significant source of micronutrients.

## Graphical abstract

This study novel analytical data of 24 commonly consumed marine fish species from East Africa to assess their nutrient content and contribution to Codex nutrient reference values. Results showed that small fish species, typically eaten whole, are richer in key micronutrients compared to larger, filleted species. These findings highlight the nutritional importance of small fish and support updates to regional food composition tables.

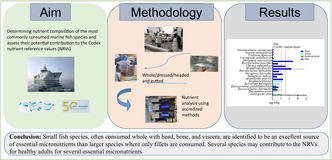

## 1 Introduction

Fish and fishery products are important sources of nutrients, such as proteins, omega‐3 polyunsaturated fatty acids, vitamins, and minerals, essential to human health. Nutritional benefits of seafood are crucial in securing health and preventing chronic diseases (Food and Agriculture Organization 2024; Golden et al. 2021). However, neither a single food nor a food group provides all the nutrients essential for good health. Seafood, however, is a valuable and well‐balanced source of several key nutrients that are otherwise challenging to obtain from other foods (Liu and Ralston 2021; Sobral et al. 2018). Besides being rich sources of protein and omega‐3 fatty acids, fish, especially small pelagic fish consumed whole with bones, head and viscera, are rich in micronutrients such as calcium, zinc, iron, iodine, vitamin A, vitamin D, vitamin B12, and folic acid (Nordhagen et al. 2020; Reksten, Somasundaram, Kjellevold, et al. 2020).

In the Western Indian Ocean (WIO) region, including Tanzania and Mozambique, fisheries play a significant role in safeguarding food and nutrition security, as well as providing income (Walmsley et al. 2006). The annual fish production in Tanzania is approximately 519,930 metric tons, with about 95% of fish sourced from small‐scale fisheries, and a per capita fish consumption of 7.17 kg/year in 2023 (Ministry of Livestock and Fisheries 2023). Regarding Mozambique, the total fish catch was estimated to be 447,000 tons in 2020, with a per capita fish consumption of 14.9 kg/year (Maulu et al. 2024). Despite relying on fisheries for more than 30% of the protein intake, the per capita fish consumption is still low compared to the global 20.7 kg/year in 2022 (Food and Agriculture Organization 2024). This limited consumption leads to a deficiency of micronutrients such as iron and vitamin B12, which are important in eliminating anemia. As a result, the prevalence of anemia is higher (> 50%) in the coastal nations of Africa compared to the coastal nations of developed countries (Viana et al. 2023). This highlights the crucial role of fish consumption in alleviating micronutrient deficiencies.

Furthermore, women of reproductive age (15–49 years) from low‐ and lower‐middle‐income countries are prone to one or more micronutrient deficiencies, such as low intake of iron, zinc or folate (Stevens et al. 2022). According to the Global Nutrition Report (2022), the prevalence of anemia in WRA was 38.9% and 47.9% in 2019 for Tanzania and Mozambique, respectively, and shows little progress in achieving the global nutrition target of 2025, which is a 50% reduction of anemia in WRA (World Health Organisation 2014). The leading cause of micronutrient deficiencies is poor dietary diversity, inherent in the high consumption of cereal‐based staple foods (Mbwana and Kinabo 2020). There is a clear link between women's micronutrient status and their children's health (Ransom and Elder 2003). Insufficient micronutrient intake, such as folic acid, iodine, zinc, and iron, for women of reproductive age before conception and during pregnancy increases the chance of preterm births or infants with neural tube defects, cretinism or low birth weights (Gernand et al. 2016). Hence, focusing on women's nutritional status is crucial because healthy women can undertake multiple roles, including securing family nutrition and their children's health (Ghosh et al. 2021).

Fish and fishery products are often undervalued in global food systems' efforts to achieve food and nutrition security (Koehn et al. 2022). Different studies have investigated the potential value of small pelagic fish species in tackling micronutrient deficiencies (Byrd et al. 2021; Hasselberg et al. 2020; Kolding et al. 2019). Small fish consumed whole with head and bones could significantly reduce micronutrient deficiencies (Nordhagen et al. 2020; Reksten, Somasundaram, Kjellevold, et al. 2020). Furthermore, fish improve the bioavailability of iron and zinc from cereal and tuber‐based foods (Consalez et al. 2022). Therefore, including a small amount of fish in the diet may provide essential nutrients for WRA (Hicks et al. 2019).

Despite its potential for improved food and nutrition security along the East African coast, there is currently insufficient data on the nutritional composition of commonly consumed marine fish species (Obiero et al. 2019). Food composition data (FCD) are the foundation of most nutritional guidelines and programs and should receive more attention in food security policies (World Health Organisation 2022). Relevant, reliable, and up‐to‐date FCDs are of fundamental importance in nutrition, dietetics, and health. They can be used in establishing dietary guidelines, dietetic research, assessing nutrient requirements, and government policy development and implementation (Greenfield and Southgate 2003). Due to the variations of nutrients in food from different countries as a factor of environment, genetics, processing, food diversity, and consumption patterns, country‐specific nutrient data are desired (Food and Agriculture Organization 2024). The Tanzania and Mozambique FCDs were established by borrowing data from other countries' databases, such as the United States Department of Agriculture National Nutrient Database (USDA), World Food Dietary Assessment System (WFDAS), South Africa, West Africa, Egypt, Kenya, India, Mali, and Senegal (Korkalo et al. 2011; Lukmanji et al. 2008). However, these FCDs include only a small fraction of the fish commonly consumed in these countries. In the case of Tanzania, all fish are grouped as either small fish from freshwater or marine water, or as fish in general, whereas the nutrient content varies between and within species (Hicks et al. 2019). This study aims to quantify the nutritional content of commonly consumed fish species from the marine waters of Tanzania and Mozambique, considering local consumption practices such as whole fish, dressed, headed and gutted, and fillets. Further, it aims to assess the contribution to food and nutrition security by estimating their contribution to the Codex nutrient reference values (NRVs) of selected micronutrients for healthy adults (Lewis 2019).

## 2 Materials and Methods

This paper uses data collected through scientific surveys with the R/V Dr. Fridtjof Nansen (Nansen survey) as part of the collaboration between the EAF‐Nansen Programme, Tanzania Fisheries Research Institute (TAFIRI), and the National Institute of Fisheries Research (IIP) of Mozambique in 2018 and 2023. The EAF‐Nansen Programme is a collaboration between the Food and Agriculture Organization of the United Nations (FAO), the Norwegian Agency for Development Cooperation (NORAD) and the Institute of Marine Research (IMR), Norway, for sustainable management of the fisheries of partner countries (Food and Agriculture organisation 2019).

### 2.1 Fish Sampling and Handling

Sampling was conducted during a survey of Tanzania and Mozambique territorial waters by R/V Dr. Fridtjof Nansen, starting from the south coast of Mozambique (−26.53S, 32.56E) to the north coast of the Tanzania mainland and Zanzibar Island (−6.00S, 35.00E). The 2018 survey was conducted from the 12th of February to the 4th of April in Mozambique and from the 6th of April to the 18th of April in Tanzania. The 2023 survey in Tanzania was carried out from the 28th of June 2023 to the 25th of July 2023 (samples not collected in Mozambique). During sampling, pelagic and demersal trawls were continuously unloaded on deck, the catch was sorted, and the fish were identified by taxonomists onboard the vessel. Species selection was based on a list of prioritized species commonly consumed in Tanzania and Mozambique (Appendix 1). Samples were prepared based on how the fish is commonly consumed in local diets. To determine the consumption habits of each species, we use a combination of approaches: direct observation at markets and landing sites, informal discussion with fishers, processors, vendors and consumers during reconnaissance surveys and local authors' experience with the local communities along the coast. Small fish were prepared as whole, dressed or headed and gutted, while large fish were prepared as a fillet. This study defines fish with head, tail, skin, viscera and bones as “*whole*,” fish without a head, viscera, and tail as “*dressed*,” fish without head and viscera as “headed and gutted,” and fish fillet as “*fillet*.” Furthermore, this study defines small fish as “fish with a maximum length of 25cm” and large fish as “fish with a length greater than 25 cm” (Bavinck et al. 2023).

The fish preparation method applied to different samples is shown in Table 1. For each species, pooled samples (composite) were prepared, with each composite sample consisting of more than 25 randomly selected individuals for the small fish and five randomly selected individuals for the large fish. From the 2018 survey, samples were homogenized and prepared wet and freeze‐dried before shipment to the IMR laboratories for further analysis, as described by (Reksten, Bøkevoll, Frantzen, et al. 2020). From the 2023 survey, the samples were packed in plastic bags and stored in insulated boxes in the vessel's freezer at −20°C until shipment by air cargo to the IMR laboratories in Bergen, Norway. After arrival at the IMR, fish samples were homogenized and prepared as wet, freeze‐dried, and stored at −80°C prior to analyses.

**TABLE 1:** Overview of fish species (scientific, English, and local names), habitat characteristics, average length (cm), and weight (g) sampled during the 2018 and 2023 R/V Dr. Fridtjof Nansen surveys along the coast of Tanzania and Mozambique.

Scientific name	Sampling year	Sampling coordinates	English name	Local name	Habitat	Tissue analyzed	*n*	*N*	Average weight (g)	Average length (cm)
Tanzania										
Small fish										
*Decapterus kurroides*	2018	−10.19S, 40.19E	Redtail scad	Lungu/Kibua macho	Reef‐associated	W	1	25	14.6	11
*Encrasicholina heteroloba*	2018	−8.68S, 39.43E	Shorthead anchovy	Uono	Pelagic	W	2	100	2.8 ± 0.1	7.3 ± 0.4
*Spratelloides gracilis*	2018	−8.18S, 39.55E	Silver‐stripe round herring	Dagaa msumari/dagaa chuma	Pelagic	W	2	150	2.0 ± 0.3	6.3 ± 0.4
*Upeneus taenopterus*	2018	−7.56S, 39.72E	Finstripe goatfish	Mkundaji	Pelagic	W	2	25	25.6 ± 2.7	12.0 ± 0.7
*Encrasicholina punctifer*	2018	−6.57S, 39.72E	Buccaneer anchovy	Dagaa –Mcheli	Pelagic	W	1	300	3	5.5
*Decapterus macrosoma*	2018	−5.75S, 39.10E	Shortfin scad	Simsimu	Pelagic	W	1	25	52.2	16.5
*Carangoides malabaricus*	2018	−5.93S, 39.15E	Malabar trevally	Kolekole	Pelagic	W	1	25	52.5	13.4
*Amblygaster sirm*	2023	−8.68S, 39.42E	Spotted sardinella	Dagaa damu/saladini	Reef‐associated	D	1	25	11.24	11.2
*Dussumieria acuta*	2023	−8.68S, 39.42E	Rainbow sardine	Dagaa simu/papa	Pelagic	D	2	11,14^b^	25.7 ± 12.6	14 ± 2.6
*Encrascicholina intermedia*	2023	−6.39S, 39.17E	Shiner anchovy	Dagaa mcheli/Dagaa nyama	Pelagic	W	3	59,60,62^b^	3.3 ± 0.9	7.6 ± 0.4
*Encrascicholina intermedia*	2023	−6.39S, 39.17E	Shiner anchovy	Dagaa mcheli/Dagaa nyama	Pelagic	H&G	3	63,66,66^b^	3.2 ± 0.9	7.3 ± 0.4
*Encrasicholina pseudoheteroloba*	2023	−6.38S, 39.03E	Shorthead anchovy	Dagaa mcheli/dagaa nyama	Pelagic	W	3	60	3.6 ± 0.7	7.7 ± 0.4
*Encrasicholina pseudoheteroloba*	2023	−6.38S, 39.03E	Shorthead anchovy	Dagaa mcheli/dagaa nyama	Pelagic	H&G	3	65,66,69^b^	3.5 ± 0.8	8.0 ± 1.0
*Restrelliger karnaguta*	2023	−5.93S, 39.09E	Indian mackerel	Kibua maji	Pelagic	D	3	9,25,32^b^	29.2 ± 34.6	14.9 ± 5.1
*Sardinella gibossa*	2023	−5.98S, 39.14S	Goldstripe sardinella	Dagaa papa/Upapa	Pelagic	D^b^	2	12,15^b^	22.1 ± 1.5	11.8 ± 1.5
*Spratelloides gracilis*	2023	−5.97S, 39.14E	Silverstripe herring	Dagaa msumari/dagaa chuma/uono	Pelagic	W	2	25,125^b^	2.2 ± 0.04	6.6 ± 0.2
*Stolephorus indicus*	2023	−8.40S, 39.67E	Indian anchovy	Dagaa‐mcheli	Pelagic	W	3	12	19.3 ± 5.2	13.7 ± 1.3
*Stolephorus indicus*	2023	−8.40S, 39.67E	Indian anchovy	Dagaa‐mcheli	Pelagic	H&G	3	13	19.3 ± 4.3	13.6 ± 1.0
Large fish										
*Trichiurus lepturus* ^a^	2018	−10.09S, 40.05E	Largehead hairtail	Mkonge‐kuti	Benthopelagic	F	5	5	624.1 ± 40.8	102.9 ± 2.5
*Saurida undosquamis*	2018	−8.17S, 39.69E	Brushtooth lizardfish	Kirukia	Reef‐associated	F	6	5	381.1 ± 24.0	37.1 ± 0.7
*Scomberomorus commerson*	2018	−5.45S, 39.68S	Narrow‐barred Spanish mackerel	Nguru‐maskati	Pelagic	F	2	5,3^b^	2497.5	76.4
Mozambique										
Small fish										
*Decapterus russelli* ^a^	2018	−25.90S, 33.00E	Indian scad	Carapau do índico	Benthopelagic	W	6	25	14.8 ± 3.1	11.7 ± 0.8
*Decapterus russelli* ^a^	2018	−25.90S, 33.00E	Indian scad	Carapau do índico		D	6	25	14.8 ± 3.1	11.7 ± 0.8
*Ommastrephes bartramii* ^a^	2018	−21.20S, 35.50E	Neon flying squid	NA		W	6	25	13.9 ± 2.4	9.0 ± 0.56
*Ommastrephes bartramii* ^a^	2018	−21.20S, 35.50E	Neon flying squid	NA		D	6	25	13.9 ± 2.4	9.04 ± 0.57
*Upeneus japonicas*	2018	−26.15S, 37.02E	Japanese goatfish	NA	Pelagic	W	3	25	24	NA
*Upeneus japonicas*	2018	−26.15S, 37.02E	Japanese goatfish	NA		D	3	25	24	NA
*Upeneus taeniopterus*	2018	−17.81S, 37.79E	Finstripe goatfish	Salmonete estriado	Pelagic	W	3	25	31	12.9
*Upeneus taeniopterus*	2018	−17.81S, 37.79E	Finstripe goatfish	Salmonete estriado		D	3	25	31	12.9
*Decapterus macrosoma*	2018	−18.51, 37.02E	Shortfin scad	Carapau barbatana	Pellagic	W	3	25	8.32	10.4
*Decapterus macrosoma*	2018	−18.51, 37.20E	Shortfin scad	Carapau barbatana		D	3	25	8.32	10.4
*Saurida undosquamis* ^a^	2018	−25.25S, 33.82E	Brushtooth lizardfish	NA	Reef‐associated	W	6	25	71.5 ± 68.5	19.3 ± 5.7
*Saurida undosquamis* ^a^	2018	−25.25S, 33.82E	Brushtooth lizardfish	NA		D	6	25	71.5 ± 68.5	19.4 ± 5.7
*Engraulis capensis*	2018	−25.06S, 34.15E	Southern African anchovy	NA	Pellagic	W	3	25	32.9 ± 1.6	15.9
*Engraulis capensis*	2018	−25.06S, 34.15E	Southern African anchovy	NA		D	3	25	32.9 ± 1.6	15.9
Large fish										
*Polysteganus coeruleopunctatus*	2018	−26.77S, 32.97E	Blue‐skin seabream	NA	Demersal	F	3	5	788.4	34.9
*Merluccius paradoxus*	2018	−26.24S, 33.41E	Deep‐water cape hake	NA	Demersal	F	3	5	469.8 ± 71.2	38.7 ± 1.7
*Pomadasys kaakan*	2018	−17.29S, 38.53E	Javelin grunter	Peixe pedra	Reef‐associated	F	2	NA	1963.2	90.5
*Scomberomorus commerson* ^a^	2018	−24.81S, 34.76E	Narrow‐barred Spanish mackerel	Serra	Pelagic	F	4	5	2866.2 ± 948.4	78.8 ± 4.0

Abbreviations: D, dressed—head, viscera and tail not included; F, fillets only included; H&G, headed and gutted‐head and viscera not included; *n*, number of composite samples; *N*, number of individual fish per composite sample; NA, not available; W, whole—head, viscera and tail included in the analysis.

^a^ Fish were sampled from two locations. Weight and length measurements are expressed as the mean of one pooled sample consisting of n number of fish.

^b^ Each pooled sample had a different number of individuals.

### 2.2 Analytical Methods

Analyses of proximate composition, vitamins, minerals and fatty acid profiles were carried out in parallel at the IMR laboratories in Norway. All procedures used to determine crude protein, crude fat, dry matter, fatty acids, vitamins and minerals are described by Reksten, Bøkevoll, Frantzen, et al. (2020).

### 2.3 Determination of Crude Protein, Fat, Dry Matter and Fatty Acid Composition

Crude protein was determined following the accredited method by the Association of Official Agricultural Chemists (AOAC 1995), and calculated from total nitrogen, determined by burning the material in pure oxygen gas in a combustion tube (Leco FP 628, Leco Corporation, Saint Joseph, MI, USA) at 950*°*C. Nitrogen was detected with a thermal conductivity detector (TCD, Leco Corporation, Saint Joseph, MI, USA), and the nitrogen content was calculated from an estimated average of 16% nitrogen per 100 g protein. Crude fat was determined by the method accredited with ISO‐EN 17025 and standardized as a Norwegian Standard, NS 9402 (Norwegian Standard 9402 1994). Crude fat was extracted with ethyl acetate and filtered before the solvent evaporated, and the fat residue was weighed. This method is preferred for non‐polar lipids (triglycerides, steryl esters) in fish, as fish lipids typically consist of 82%–98% non‐polar lipids (Lambertsen 1972). The dry matter of the samples was determined by the freeze‐drying method. Samples were weighed first before freeze drying for 72 h (24 h at −20°C, then followed by 48 h at +25°C, with a vacuum of 0.2–0.01 mbar, Labconco Freezone 18 L mod. 775030, ID no: 3311). Samples were then weighed and recorded immediately. The weight difference of the sample before and after freeze drying was used to calculate the dry mass of the sample by using the following formula

$$\%\mathrm{Dry}\;\text{matter}=\frac{\left(c-d\right)}{\left(a-b\right)}\times 100$$

where: *a* = Initial weight of sample + container (g), *b* = Initial weight of container (g), *c* = Final weight of the sample + container (g) and *d* = Final weight of container (g). For fatty acids determination, lipids from the samples were extracted according to (Folch et al. 1957). The fatty acid composition was analyzed using a gas–liquid chromatograph (GLC) as described by (Lie and Lambertsen 1991; Torstensen et al. 2004).

### 2.4 Determination of Vitamins and Minerals

The samples were saponified and extracted to determine vitamin A1 (sum all‐trans retinol and 13‐, 11‐, 9‐cis retinol) and A2 content. Vitamin A1 and A2 were determined by high‐performance liquid chromatography (HPLC) (normal phase) using a Photo Diode Array (PDA) detector, and the retinol content was calculated by external calibration standard curve as previously described in (Comitè Europèen de Normalisation 2000). For the determination of vitamin B12 (cobalamin), microorganisms (*Lactobacillus delbruecki*i –ATCC 4797) were added and incubated at 37°C for 22 h (Angyal and Food and Drug Administration 1996). Meanwhile, to determine vitamin B9, the microorganism *Lactobacillus rhamnosus* (ATCC 7469) was added and incubated at 37°C for 20 h. The vitamin content was calculated by comparing the growth of the organism in the unknown samples with the growth of the organism in known standard concentrations by turbidimetric reading (AOAC 1996). The concentrations of minerals (Fe, Zn, I, and Ca) were determined by Inductively Coupled Plasma‐Mass Spectrometry (iCapQ ICPMS, ThermoFisher Scientific, Waltham, MA, USA) equipped with an auto‐sampler (FAST SC‐4Q DX, Elemental Scientific, Omaha, NE, USA) after wet digestion in a microwave oven (UltraWave, Milestone, Sorisole, Italy), as described by Julshamn et al. (2001) and Julshamn et al. (2007).

### 2.5 Data Management and Presentation of Data

All analytical values of nutrients were recorded in the Laboratory Information Management System (LIMS) and exported to Microsoft Excel 2016 for further statistical analysis. The data for all nutrient contents are presented on graphs as means and standard deviations (SD), and Supporting Information tables present the nutrient values as means and SD with the same unit of expression as advised by FAO guidelines for FCD (Greenfield and Southgate 2003). The values for protein and fat were presented as g/100 g ww (wet weight). Vitamin A was presented as μg/100 g isomers retinol (the sum of 13‐, 11‐, 9‐cis and all‐trans‐retinol (A1) and dehydro all‐trans‐retinol (A2)). The vitamin A2 values that were below the limit of quantification (LOQ = 0.05 μg/100 g) were presented as < 0.05 μg/100 g. Iron, zinc and calcium were in mg/100 g, whereas iodine was in μg/100 g. The data for EPA and DHA were presented in g/100 g and reported as a percentage of the total fatty acids. Statistical analysis was performed using R statistical software (R 4.3.2) operated in R‐Studio (version 1.3.959). When the data did not meet the normality assumption, they were log‐transformed. One‐way analysis of variance (ANOVA), accompanied by multiple post hoc pairwise comparisons at a significance level of 0.05, was used to assess the differences in nutrients between the species and the tissue analyzed. A principal component analysis (PCA) was performed using the “FactoMineR” package in R to examine the interrelationships among nutrients and fish tissues. By projecting high‐dimensional data into a two‐dimensional space, the analysis enabled the identification of key patterns of nutrient variation and correlation. The results also revealed clusters in nutrient composition, influenced by factors such as habitat, fish size, and tissue type, thereby strengthening the interpretation of our findings in the context of food security. Pearson correlation coefficients were also calculated to identify the correlation patterns among nutrients.

### 2.6 Calculation of Contribution to Codex Nutrient Reference Values

The contribution of each species to the NRVs of vitamin A, folic acid, vitamin B12, calcium, iron, iodine, and zinc was calculated with reference to Codex nutrient reference values–requirements (NRVs) of healthy dietary intake for the general population (Lewis 2019). The NRVs for vitamin A, folic acid, vitamin B12, calcium and iodine were 800 μg/day, 400 μg/day, 2.4 μg/day, 1000 mg/day and 150 μg/day, respectively. For iron, the NRVs of 14 and 22 mg/day were used, considering 15% and 10% dietary absorption, respectively, and for zinc, 11 and 14 mg/day with 30% and 22% dietary absorption, respectively. For EPA + DHA, the adequate intake (AI) of 250 mg/day from the European Food Safety Authority (European Food Safety Authority 2017) for healthy adults was used. All NRVs were assessed using an estimated 100 g portion of raw fish. A fish species is defined as a *good source* of a specific nutrient if it contains 15% to 30% of an NRV/AI per 100 g of edible portion, and will be considered an *excellent source* of this nutrient if it contains more than 30% of a particular nutrient per 100 g edible portion.

## 3 Results

### 3.1 Sample Characteristics

This study includes 123 pooled samples (composite) of 24 marine fish species sampled during the Nansen surveys along the coast of Tanzania and Mozambique in 2018 and 2023. Table 1 presents an overview of each species' identification details, scientific name, common name, local names when available, habitat, weight, and length.

### 3.2 Nutrient Composition of Fish Species Sampled From Marine Waters of Tanzania and Mozambique During the Nansen Survey of 2018 and 2023

**FIGURE 1:**
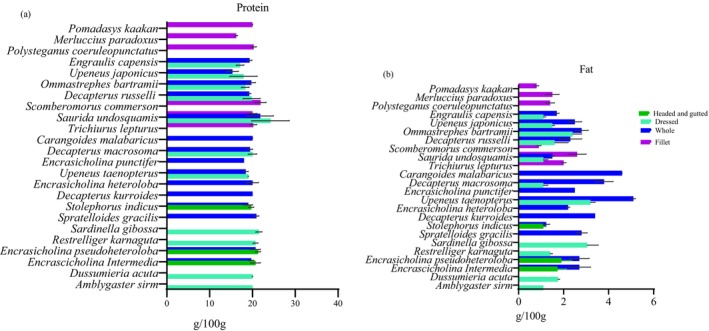
Protein (a) and fat (b) (g/100 g) content of fish species sampled from coastal waters of Tanzania and Mozambique during the R/V *Dr. Fridtjof Nansen* surveys of 2018 and 2023. Values are presented as means ± standard deviations (SD) of the fish species analyzed and expressed as the nutrient content per 100 g raw, edible part.

**FIGURE 2:**
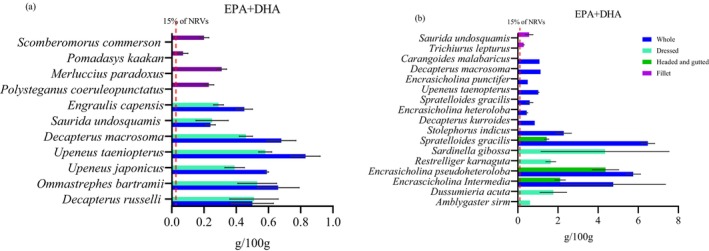
The content of EPA + DHA (g/100 g) in fish species sampled from coastal water of (a) Mozambique and (b) Tanzania during the R/V Dr. Fridtjof Nansen surveys of 2018 and 2023. Values are presented as means ± standard deviations (SD) of the fish species analyzed and expressed as the nutrient content per 100 g raw, edible part. The dashed line shows the species that meet a 15% contribution to the adequate intake (AI) for the EPA + DHA of healthy adults.

**FIGURE 3:**
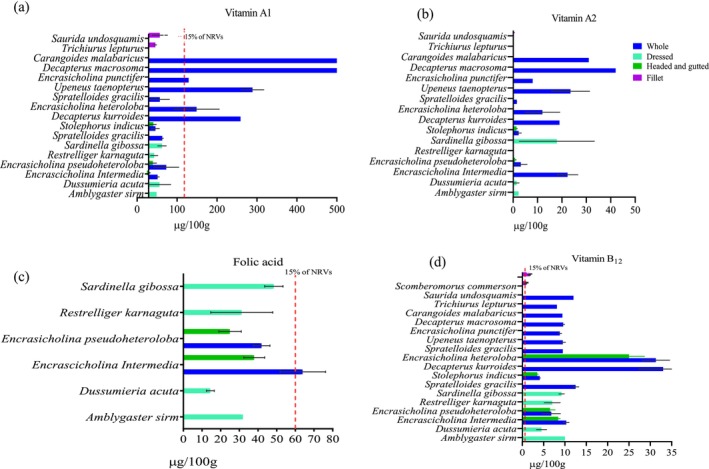
The content of (a) Vitamin A1, (b) vitamin A2, (c) folic acid, and (d) vitamin B12 in fish species sampled from coastal water of Tanzania during the R/V Dr. Fridtjof Nansen survey of 2018 and 2023. Values are presented as means ± standard deviations (SD) of the fish species analyzed and expressed as the nutrient content per 100 g raw, edible part. The dashed line shows the species that meet a 15% contribution to nutrient requirement value (NRV) for the particular vitamin.

**FIGURE 4:**
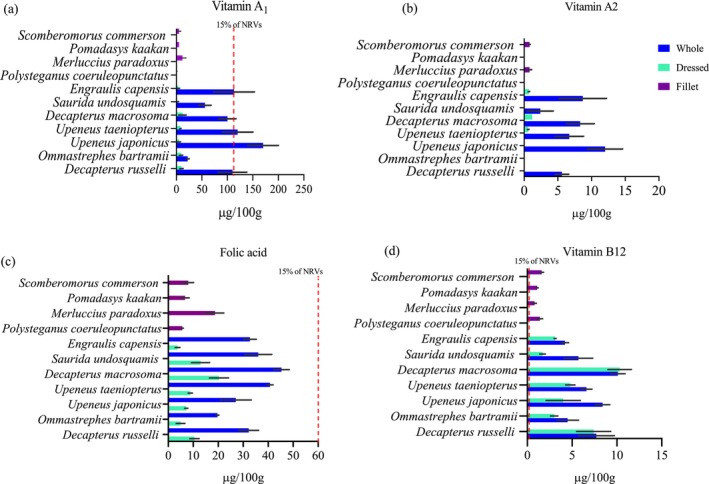
The content of (a) vitamin A1, (b) vitamin A2, (c) folic acid, and (d) vitamin B12 in fish species sampled from coastal waters of Mozambique during the R/V Dr. Fridtjof Nansen survey of 2018. Values are presented as means ± standard deviations (SD) of the fish species analyzed and expressed as the nutrient content per 100 g raw, edible part. The dashed line shows the species that meet a 15% contribution to nutrient requirement value (NRV) for the particular vitamin.

**FIGURE 5:**
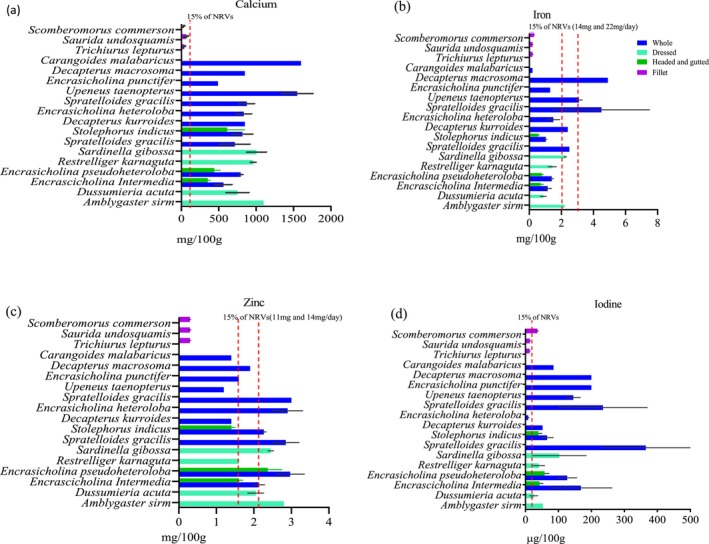
The content of (a) calcium, (b) iron, (c) zinc, and (d) iodine in fish species sampled from coastal waters of Tanzania during the R/V Dr. Fridtjof Nansen surveys of 2018 and 2023. Values are presented as means ± standard deviations (SD) of the fish species analyzed and expressed as the nutrient content per 100 g raw, edible part. The dashed line shows the species that meet a 15% contribution to nutrient requirement value (NRV) for the particular mineral. For iron, the NRV of 14 and 22 mg/day was used considering 15% and 10% dietary absorption, respectively, and for zinc, 11 and 14 mg/day with 30% and 22% dietary absorption, respectively.

**FIGURE 6:**
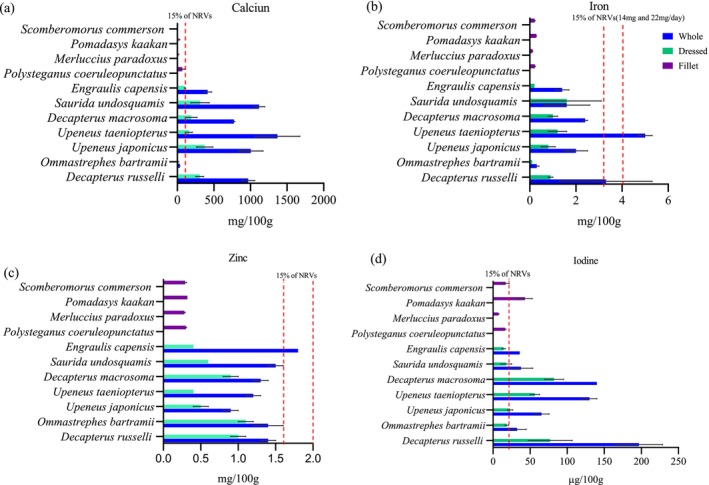
The content of (a) calcium, (b) iron, (c) zinc, and (d) iodine in fish species was sampled from coastal water in Mozambique during the R/V Dr. Fridtjof Nansen survey of 2018. Values are presented as means ± standard deviations (SD) of the fish species analyzed and expressed as the nutrient content per 100 g raw, edible part. The dashed line shows the species that meet a 15% contribution to nutrient requirement value (NRV) for the particular mineral. For iron, the NRV of 14 and 22 mg/day was used considering 15% and 10% dietary absorption, respectively, and for zinc, 11 and 14 mg/day with 30% and 22% dietary absorption, respectively.

The nutrient content of different fish species is presented in Figures 1, 2, 3, 4, 5, 6. Protein content ranged from 15 ± 1.0 g/100 g to 24 ± 1.4 g/100 g and did not significantly differ between tissues analyzed or between species (*p* > 0.05). Fat content was significantly higher in whole and dressed small fish compared to fillet samples of large fish (*p* < 0.05) (Table S1). In general, all fish species had a higher content of unsaturated fatty acids than saturated fatty acids. Nevertheless, there was no significant difference in fatty acid composition between the fish species analyzed whole, dressed, headed and gutted (*p* > 0.05). The small fish species contained higher levels of both EPA and DHA than the fillet samples, with the significantly highest level observed in *Upenius taenopterus* (*p* < 0.05) (Tables S2 and S3). The vitamins A1, A2, and folic acid were significantly higher in whole than in dressed, headed and gutted, and fillet samples (*p* < 0.05). However, vitamin B12 shows no significant difference between the tissues analyzed (*p* > 0.05). Furthermore, there were significant differences in vitamin content with higher values observed in whole samples of *Decapterus macrosomia*, *Carangoides malabaricus* , *Upenius taeniopterus*, and *Spretelloides gracilis* (*p* < 0.05) (Table S4). The mineral content varied considerably between the tissues analyzed and whole fish samples contained significantly higher amounts of calcium, iron, iodine and zinc than the fillet and dressed samples (*p* < 0.05) (Table S4).

### 3.3 Correlation Between Nutrients and Fish Tissues Analyzed

**FIGURE 7:**
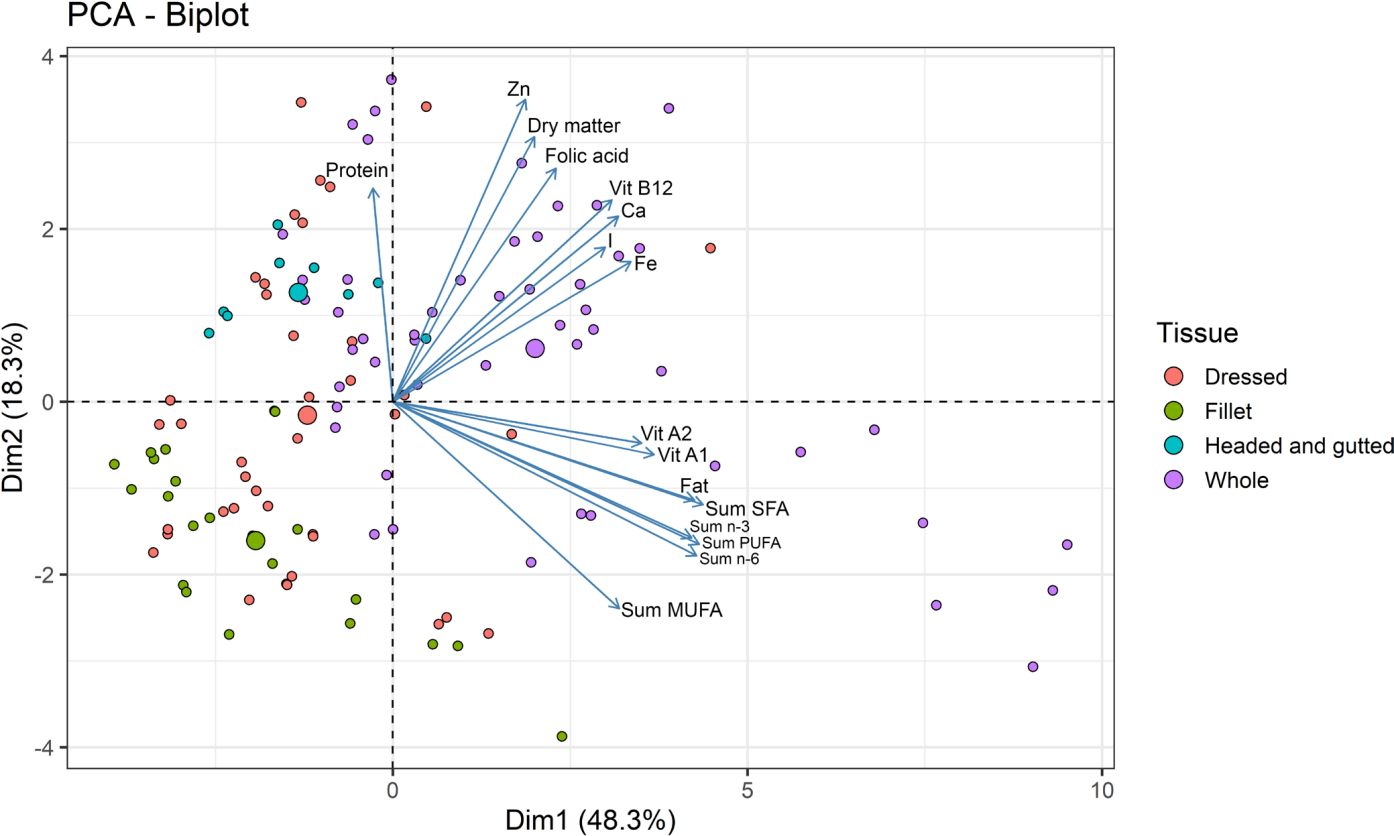
Principal component analysis (PCA) for nutrients in tissues fillet, whole (with head, viscera, bones, tail), and dressed fish (without head and viscera) for fish species sampled from marine waters of Tanzania and Mozambique.

The two‐dimensional projection in the PCA analysis captured the majority of the variation in the data, with the first principal component explaining 48.3% and the second 18.3% of the total variability (Figure 7). Omega‐3 fatty acids, vitamins A1 and A2 and fat were positively correlated to some extent, as their vectors appeared in a similar direction. This observation was supported by the correlation matrix (Figure S1), which showed a moderate to strong positive association between omega‐3 fatty acids, vitamins A1 and A2, and fat content (*r* = 0.54 to 0.88). Species with high fat content, such as *Upenius taenopterus*, *Decapterus macrosoma* and *Carangoides malabaricus* , also showed higher content of omega‐3 fatty acids and vitamins A1 and A2 (Figure S2, Tables S2 and S3). Additionally, micronutrients, including calcium, iron, iodine, and zinc, vitamin B12, and folic acid, which are highly water‐soluble, were positively correlated with each other. Moderate correlations between these nutrients confirmed this in the correlation matrix (*r* = 0.40 to 0.67). The results also showed that whole small fish bear the highest levels of these micronutrients, followed by dressed, headed and gutted, while large fish fillets showed the lowest levels (Tables S4 and S5).

### 3.4 Potential Contribution to the Codex Nutrient Reference Values

The potential contribution of different fish species to the NRVs of calcium, iron, zinc, vitamin A, EPA and DHA is presented in Figures 1, 2, 3, 4, 5, 6. Most of the whole fish samples contribute ≥ 50% of the daily NRV of calcium, and are shown to contribute ≥ 100% of the daily NRV of vitamin B12 and EPA + DHA when 100 g of fish is consumed. All fillet samples contributed less than ≤ 10% of the daily NRV of calcium, iodine, zinc and folic acid. The majority of small fish species analyzed whole with head, viscera, and tail will contribute ≥ 15% of NRV for vitamin A when 100 g of fish is consumed. However, all fillets of large fish, dressed and headed small fish, contribute less than < 10% of NRV for vitamin A when the same amount is consumed.

## 4 Discussion

This paper presents the nutrient composition of 24 commonly consumed fish species from the coastal waters of Tanzania and Mozambique during the Nansen survey of 2018 and 2023, as presented in Table 1. The data set represents the diversity of pelagic, reef‐associated and demersal species with different sizes and weights that reflect the nutrient data presented in this study, capturing the wide range of ecological niches and feeding habits that influence the nutrient content in fish. Moreover, it is important to note that some species, such as *Upeneus japonicas* and *Pomadays kaakan*, have NA values for average height and number of individual samples in pooled samples (*N*). As the samples were collected onboard research vessels, some morphometric measurements may not be recorded due to logistical challenges such as rigorous sampling periods and handling of large catches. However, the unreported values may not alter the nutrient content, as the standardized edible tissues were only used.

To our knowledge, this is the first study that documents a wide array of chemically analyzed nutrients for the varieties of marine fish species commonly consumed on the eastern coast of Africa. In addition, this study enables the comparison of the contribution of nutrients associated with differences in household preparation methods or when the whole or dressed fish, as opposed to only the fillet, is consumed from small and large fish species, respectively. All species are considered good sources of nutrients such as protein, vitamins, minerals, and the fatty acids EPA and DHA. However, whole small fish species, consumed with head, viscera, and bones, contribute significantly higher amounts of micronutrients compared to large fish species, where only the fillet is consumed or dressed small fish, where the head, viscera, and tails are removed.

Several small fish species consumed whole were shown to be good or excellent sources of micronutrients and may contribute higher proportions to the NRVs of Vitamin A, B12, calcium, iodine, zinc, iron and AI of EPA + DHA. Only a small number of studies have been conducted to study the nutrient composition of marine fish species in Tanzania (Masanja et al. 2021; Mwakaribu et al. 2023; Shija 2019). However, they all covered a limited range of species and nutrients. Among the species reported in this study, only one species (Indian mackerel) is included in the Mozambican FCD but not the Tanzanian FCD. Thus, the results from this study may contribute significantly to the revision of current FCDs as they cover data from locally harvested fish species with a variety of small and large fish species, as well as the impact of preparation methods.

### 4.1 Proximate Composition

This study's results show no significant difference in protein content between the fish of different sizes or whether the fish was dressed or filleted. These results are consistent with Reksten, Somasundaram, Kjellevold, et al. (2020) on the contents of nutrients in fish species from Sri Lanka. All species contain more than 15% protein, and a portion of 100 g can contribute to between 30% and 50% of the daily protein requirements in the diet (World Health Organization, and United Nations University 2007). The protein in fish is primarily found in the larger muscle tissues, which could explain why both whole fish and fillets bear similar protein levels (Ochiai and Ozawa 2020). Regarding the total fat content, we found significant variations between species; small fish species (whole) had higher fat content than large fish (fillets). Fat content is species‐specific, depending on available diets and spawning season, and tends to be higher in small pelagic fish compared to large demersal fish (Ho et al. 2024; Zhu et al. 2025). Our results are comparable with those of Nordhagen et al. (2020), who found that the fat content in pelagic and mesopelagic fish ranges from 1.3 to 3.3 g/100 g. However, for the demersal fish, the fat content was lower (0.9 g/100 g) compared to that of demersal species *Saurida undosquamis, Trichiurus lepturus, Polysteganus coeruleopunctatus* and *Merluccius paradoxus* in this study. In general, the fat content is higher in this study, with the highest value of 4.9 g/100 g in whole *Upenius taenopterus* than in the study of fish species from Sri Lanka by Reksten, Somasundaram, Kjellevold, et al. (2020), who reported the highest fat content of small fish ( *Decapterus macrosoma* ) to be 2.7 g/100 g. The variation in fat content may be attributed to various factors such as species, diet, age, sex, season and climate, as described by (Nava et al. 2023).

### 4.2 Fatty Acid Composition

The finding of this study shows that both fish samples contain a considerable amount of omega‐3 fatty acids, with the highest levels found in small fish species consumed whole. The high content of EPA and DHA in small fish may be attributed to feeding habits, such as feeding on plankton and algae rich in omega‐3 fatty acids (Jónasdóttir 2019). Also, inclusion of head, bones and viscera where omega‐3 fatty acids are concentrated. The highest values of EPA and DHA in whole small fish presented in this paper (0.32 and 0.87 g/100 g, respectively) are higher than those reported in the FAO/INFOODS database of 0.09 and 0.19 g/100 g in anchovies (Ruth Charrondière et al. 2013). The levels are also higher than those of (Reksten, Somasundaram, Kjellevold, et al. 2020), who reported the highest level of EPA and DHA of 0.25 and 0.39 g/100 g, respectively, for small fish *Decapterus macrosoma* and *Auxis thazard* from Sri Lanka. For the nutrition point, EPA and DHA are crucial in neural development, cardiovascular health, and vision health, especially for infants and pregnant women. The highest values of EPA and DHA of whole small fish underscore the crucial role in diversifying diets, especially in many low‐ and middle‐income countries, where other sources of omega‐3 fatty acids, such as supplements and fortified foods, are scarce and unaffordable.

### 4.3 Content of Vitamins

There was a significant difference in the vitamin A content between whole and dressed small fish or fillets of large fish. These results are consistent and comparable with the results of previous studies (Mohanty et al. 2019; Nordhagen et al. 2020; Reksten, Somasundaram, Kjellevold, et al. 2020; Sam 2021a). The high content of vitamin A in whole fish may be explained by the inclusion of the retina and viscera, as reported by (Roos et al. 2007), where more than 95% of the vitamin A was found in the retina and viscera of small fish species such as *Amblypharyngodon mola* from Bangladesh. Moreover, we report significant levels of vitamin A2 in some small fish species like *Upeneus taenopterus*; such high levels are almost exclusively documented for freshwater fish (La Frano and Burri 2014; La Frano et al. 2018). Therefore, it is crucial to analyze both isomers of vitamin A in marine species. The mean content of vitamin B12 was higher in small fish species than in large fish; however, there was no significant difference between the whole, dressed, headed and gutted small fish. Our results are comparable with those of Nordhagen et al. (2020), who reported a mean of 9.9 and 1.1 μg/100 g of small and large fish, respectively. However, the contents are lower when compared to the study of Sam (2021b), who found 12.7 and 28.3 μg/100 g for fresh sardines and anchovies, respectively. Small fish feeding on a diet richer in vitamin B12, such as plankton and small crustaceans, can lead to higher levels in their tissues (Bito et al. 2018). The whole small fish had higher folic acid (vitamin B9) contents than the dressed small fish. This may be explained by removing viscera in dressed samples, as folic acid is higher in liver and visceral tissues (Mohanty et al. 2019).

### 4.4 Mineral Content

The fillets of large fish are observed to have lower mineral concentration compared to the whole small fish. This can be attributed to the removal of bones and scales, which are the primary source of minerals in fish. Likewise, dressed fish had lower mineral content compared to whole fish; this can reflect on the removal of head, viscera and tail during processing. Including all edible fish parts in the diet is important for food and nutrition security (Bogard et al. 2015). It has been reported that about 99% of calcium is concentrated in fish's bones, teeth, and scales. Also, small fish species' soft, edible bones contain highly bioavailable calcium (Hansen et al. 1998; Malde et al. 2010; Roos et al. 2003). The trend of higher calcium, iron, iodine and zinc levels in whole small fish compared to fillets of large fish is consistent with previous studies performed in India (Mohanty et al. 2019), Northwest Africa (Aakre et al. 2020), Bangladesh (Nordhagen et al. 2020), Sri Lanka (Reksten, Somasundaram, Kjellevold, et al. 2020), and Ghana (Sam 2021b). However, variations due to different species, geographical locations, seasons, sex, age, and the condition of the fish are evident (Sara et al. 2002; Všetičková et al. 2020).

### 4.5 Correlation Between Nutrients and Fish Tissues Analyzed

The strong positive association between the fat content, vitamin A, and omega‐3 fatty acids can be explained by the lipophilic nature of these nutrients (Turchini et al. 2009). Vitamin A, especially in retinol form, is lipophilic and likely higher in fatty fish (Roos et al. 2002). This implies that consuming whole small fish species has the advantage of getting both omega‐3 fatty acids and vitamin A. On the other hand, the positive correlation among micronutrients, including calcium, iron, iodine, and zinc, vitamin B12, and folic acid, can be attributed to their co‐occurrence in fish tissues. Bones of the fish are high in calcium, iron and zinc, same as vitamin B12, folic acid and iron are concentrated in viscera and liver (Roos et al. 2007; Toppe et al. 2007). Moreover, these micronutrients have more or less the same biological functions, such as bone formation, red blood cell synthesis and cellular function, which implies that they can be found in similar food sources (Prentice et al. 2006). Consequently, whole fish are concentrated with these micronutrients, which influence their statistical association. The patterns of these nutrients highlight the advantage of consuming whole small fish, which provide overlapping nutritional benefits that can help in combating multiple micronutrient deficiencies.

### 4.6 Potential Contribution to the Nutrient Requirements of Healthy Adults

Various studies have evaluated the contribution of nutrients from the consumption of small indigenous marine and freshwater fish to the recommended daily intake (RNI) of pregnant, lactating women and children (Bogard et al. 2015), women of reproductive age (Nordhagen et al. 2020; Reksten, Somasundaram, Kjellevold, et al. 2020) and children (Sam 2021b), and found that small indigenous fish could generally contribute with > 25% of RNI for three or more micronutrients when a 100 g portion is consumed. This aligns with this study, where we calculated that the consumption of whole small fish species would increase the contribution of micronutrients compared to the consumption of large fish species. The consumption of 100 g of whole small fish species is shown to contribute more than ≥ 100% of NRV for vitamin B12, EPA + DHA, calcium, and iodine. In addition, almost half of the small fish can contribute with ≥ 15% of NRVs for vitamin A, iron, and zinc when a 100 g portion is consumed, and thus are regarded as good sources of nutrients when compared with the recommendations of the Codex NRVs (Lewis 2019). Whole small fish species are nutrient powerhouses, and regular consumption may address deficiencies in key micronutrients and contribute significantly to overall health and well‐being. Small pelagic fish species are often abundant in coastal areas and are readily accessible to local communities. Due to their abundance and relatively low market value compared to large fish species, small pelagic fish are often more affordable for consumers, especially in regions with limited economic resources (Kabahenda et al. 2011; Kawarazuka and Béné 2011). This affordability makes them accessible to a broader range of people, including those with lower incomes. Therefore, community‐based nutrition programs and initiatives may focus on promoting their consumption as part of efforts to improve local nutrition and food security, especially in developing countries (Hicks et al. 2019). Due to the variability in nutrient content between species, as presented in this paper, it is essential to analyze the nutritional quality of many species along the coast of East Africa to have accurate and reliable food composition tables that also allow considerations of inter‐species variability.

### 4.7 Strengths and Limitations

The data presented in this paper are of high quality as the samples were analyzed at a national reference laboratory using accredited methods by NS‐EN ISO/IEC 17025 standards. Therefore, the data presented in this paper may contribute to the future revision of the FCDs of Tanzania and Mozambique. Moreover, this study presents the data analyzed from different fish tissues, which provide insight into the impact of the consumption of nutrient‐dense parts, especially for small fish. Furthermore, several species were sampled from two locations, making the nutrients' mean values more accurate. However, the variation within the individual species cannot be illustrated due to the pooling of the samples. Representative sampling is the foundation of the high quality of FCD that may be used to incorporate current FCDs (Greenfield and Southgate 2003). In this study, however, for some species, only one pooled sample (composite) was analyzed, and some pooled samples contained five individual species due to the low availability of the fish species, which is lower than recommended by standards of at least ten specimens (Greenfield and Southgate 2003). Nutrient content in fish may vary seasonally due to the influence of food availability, stage of life cycle, and environmental conditions such as the temperature of the water (Rasul et al. 2021). However, this study did not account for these variations due to the nature of the survey. Pooled sampling was used to assess the nutritional content of commonly consumed fish species from the marine waters of Tanzania and Mozambique. This method helps reduce the cost of laboratory analyses but does not capture the variation that may arise from individual differences. Investigating nutrient variation patterns requires a larger dataset and higher costs associated with individual sample analysis. However, it can offer further insights into how biological and environmental (abiotic) factors influence nutrient composition.

## 5 Conclusions

This study highlights the significant nutritional contributions of 24 marine fish species from the coastal waters of Tanzania and Mozambique. The species described in this paper present higher content for most micronutrients, such as calcium, iron, zinc, folic acid, and vitamin B12, than those presented in current FCDs. Furthermore, small fish species, often consumed whole with head, bone, and viscera, are identified to be an excellent source of essential micronutrients such as calcium, iron, iodine, zinc, and vitamins A and B12 and EPA and DHA compared to larger species where only fillets are consumed. Several species may contribute several essential micronutrients to meet the NRV for healthy adults. This study suggests that prioritizing small fish in the diet, especially for women of reproductive age, could improve public health outcomes, particularly in regions prone to micronutrient deficiencies. The data presented in this study also make a valuable addition to revising FCDs of Tanzania and Mozambique and enhance the understanding of fish as a significant source of micronutrients. However, it is important to note that our results are based on the nutrient composition of raw fish, as the household preparation or further processing and handling methods could impact the contents of nutrients. Therefore, further studies should focus on the different processing methods and ready‐to‐eat dishes to better understand the nutritional profile along the whole value chain.

## Author Contributions

**Mackrina Patrick Nombo:** conceptualization (equal), formal analysis (lead), methodology (equal), validation (equal), visualization (lead), writing – original draft (lead), writing – review and editing (lead). **Betina Lukwambe:** conceptualization (equal), funding acquisition (equal), methodology (supporting), project administration (equal), supervision (lead), writing – review and editing (supporting). **Maria Wik Markhus:** conceptualization (equal), supervision (equal), writing – review and editing (equal). **Talhiya Maulid Ali:** conceptualization (supporting), writing – review and editing (supporting). **Edel O. Elvevoll:** conceptualization (equal), supervision (equal), validation (equal), writing – review and editing (equal). **Quang Tri Ho:** formal analysis (lead), visualization (equal), writing – review and editing (equal). **José Mateus Vilanculo:** writing – review and editing (supporting). **Marian Kjellevold:** conceptualization (equal), data curation (lead), investigation (equal), methodology (equal), project administration (equal), resources (equal), supervision (equal), validation (equal), writing – original draft (equal), writing – review and editing (equal).

## Conflicts of Interest

The authors declare no conflicts of interest. The funders were not involved in any way in designing the study, data collection, analyses, interpretation of the data, manuscript writing, or the decision to publish the results. The views expressed in this article are those of the authors and do not necessarily reflect the views or policies of FAO or the Government of Norway.

## Supporting information


**Figure S1:** fsn371159‐sup‐0001‐FigureS1.docx.

**Figure S2:** fsn371159‐sup‐0002‐FigureS2.docx.

**Table S1:** fsn371159‐sup‐0003‐TableS1.docx.

**Table S2:** fsn371159‐sup‐0004‐TableS2.docx.

**Table S3:** fsn371159‐sup‐0005‐TableS3.docx.

**Table S4:** fsn371159‐sup‐0006‐TableS4.docx.

**Table S5:** fsn371159‐sup‐0007‐TableS5.docx.

## Data Availability

The data that supports the findings of this study are available in Supporting Information of this article.

## Appendix 1 List of Priority Species During the 2018–2023 Nansen Survey in Tanzania and Mozambique

**Table:**

SN	Species name	English name
1	*Decapterus kurroides*	Redtail scad
2	*Trichiurus lepturus*	Largehead hairtail
3	*Encrasicholina heteroloba*	Shorthead anchovy
4	*Spratelloides gracilis*	Silver‐stripe round herring
5	*Saurida undosquamis*	Brushtooth lizardfish
6	*Upeneus taenopterus*	Striped goatfish
7	*Encrasicholina punctifer*	Buccaneer anchovy
8	*Carangoides malabaricus*	Malabar trevally
9	*Decapterus macrosoma*	Shortfin scad
10	*Scomberomorus commerson*	Narrow‐barred Spanish mackerel
11	*Polysteganus coeruleopunctatus*	Bluespotted seabream
12	*Merluccius paradoxus*	Deep‐water Cape hake
13	*Decapterus russelli*	Indian scad
14	*Ommastrephes bartrandi*	Neon flying squid
15	*Upeneus japonicus*	Japanese goatfish
16	*Pomadasys kaakan*	Javelin grunter
17	*Selar crumenophthalmus*	Bigeye scad
18	*Engraulis capensis*	Cape anchovy
19	*Dussumieria acuta*	Rainbow sardine
20	*Encrascicholina intermedia*	Shiner anchovy
21	*Encrasicholina pseudoheteroloba*	Shorthead anchovy
22	*Restrelliger karnaguta*	Indian mackerel
23	*Sardinella gibossa*	Goldstripe sardinella
24	*Stolephorus indicus*	Indian anchovy
