# Supplementary material for: Nutrient Composition of Marine Fish Species From the East African Coast: Implications for Food and Nutrition Security
Source: Food Sci Nutr. 2026 Jan 13;14(1):e71159. doi: 10.1002/fsn3.71159 (PMC12796853; doi:10.1002/fsn3.71159)
Supplement: Supplementary file 1 — Figure S1: fsn371159‐sup‐0001‐FigureS1.docx. [file FSN3-14-e71159-s006.docx]

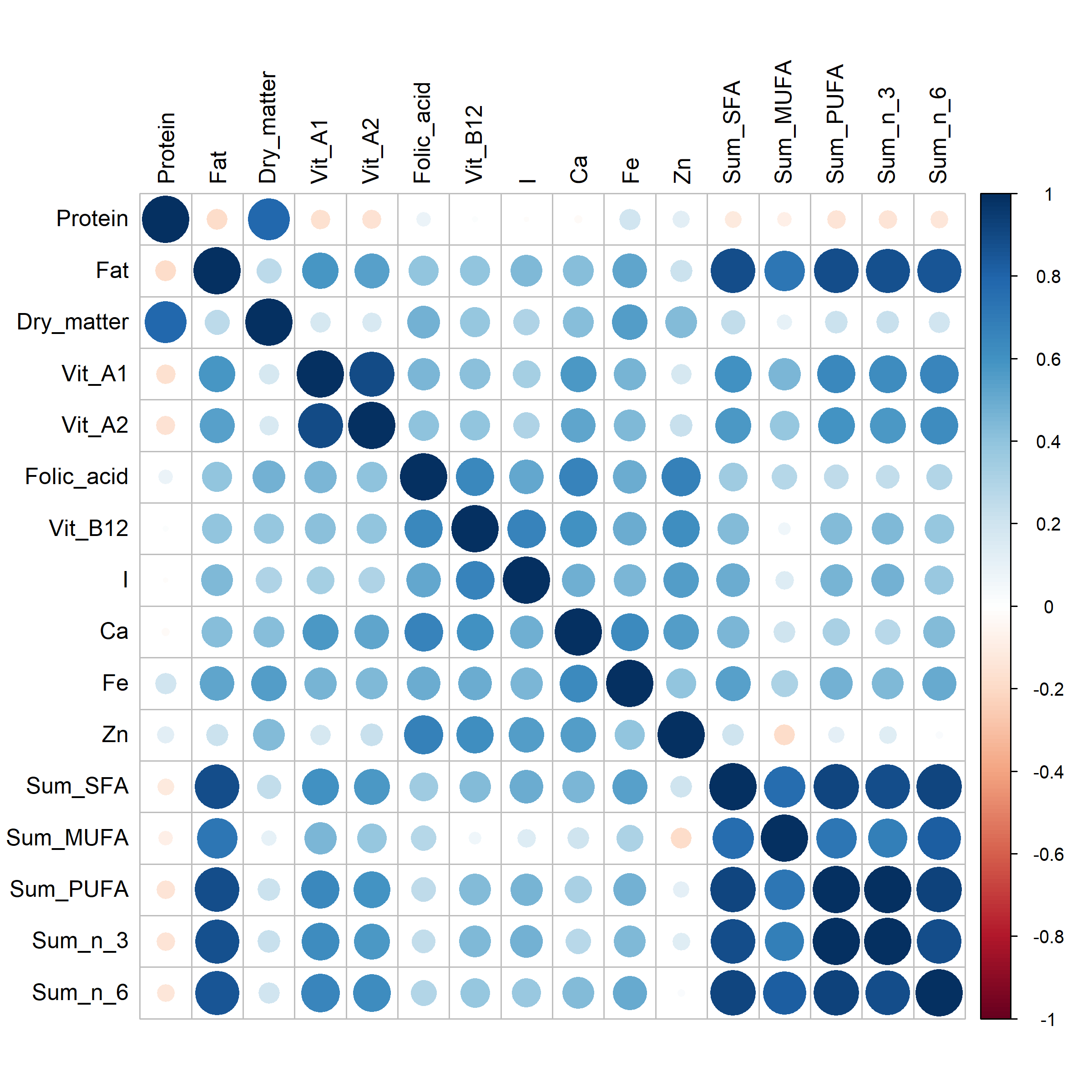


**Figure** **S1**: Correlation matrix of macro- and micronutrients across all fish species sampled from marine waters of Tanzania and Mozambique. The data were log-transformed. Colour bar and circle size indicate the strength of correlation.
