# Supplementary material for: Nutrient Composition of Marine Fish Species From the East African Coast: Implications for Food and Nutrition Security
Source: Food Sci Nutr. 2026 Jan 13;14(1):e71159. doi: 10.1002/fsn3.71159 (PMC12796853; doi:10.1002/fsn3.71159)
Supplement: Supplementary file 2 — Figure S2: fsn371159‐sup‐0002‐FigureS2.docx. [file FSN3-14-e71159-s003.docx]

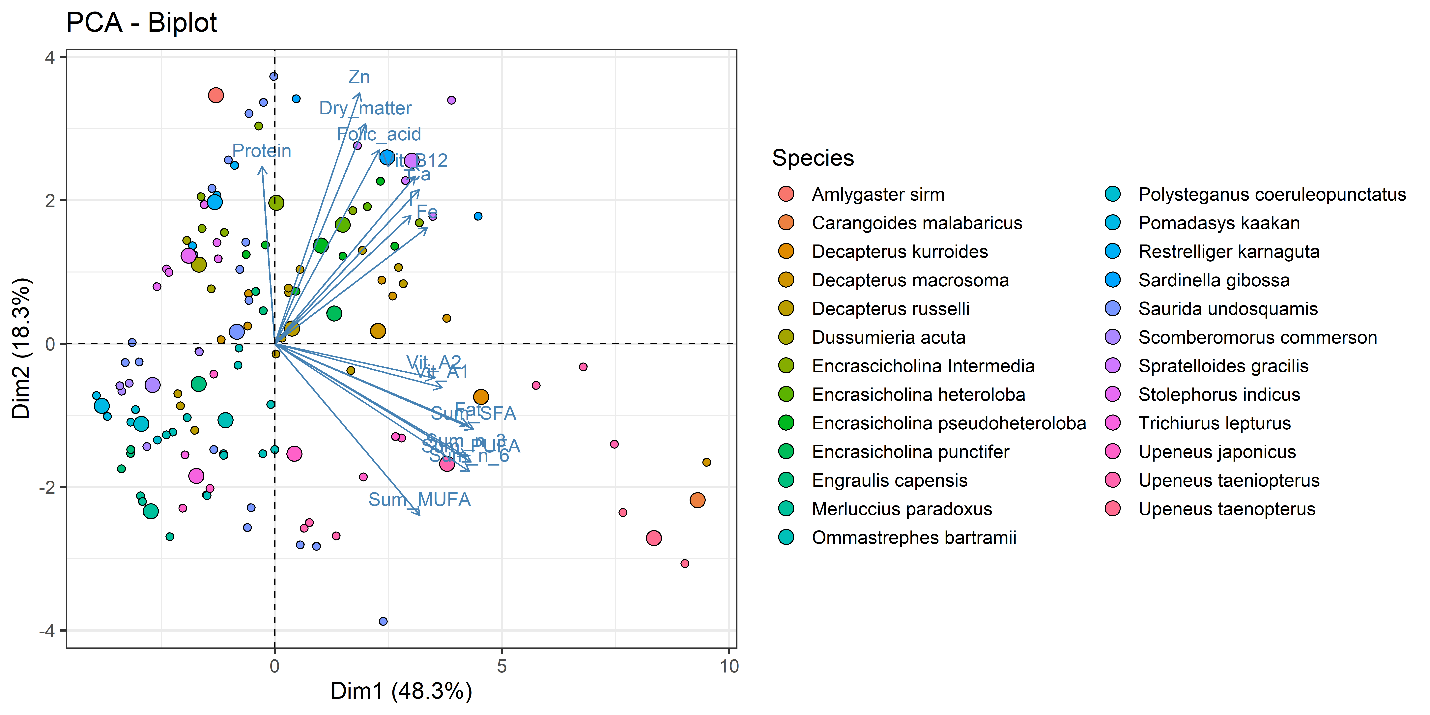


**Figure S2.** Principal Component Analysis (PCA) for nutrients in fish species sampled from marine waters of Tanzania and Mozambique.
