# Supplementary material for: Nutrient Composition of Marine Fish Species From the East African Coast: Implications for Food and Nutrition Security
Source: Food Sci Nutr. 2026 Jan 13;14(1):e71159. doi: 10.1002/fsn3.71159 (PMC12796853; doi:10.1002/fsn3.71159)
Supplement: Supplementary file 3 — Table S1: fsn371159‐sup‐0003‐TableS1.docx. [file FSN3-14-e71159-s005.docx]

**Table S1:** Proximate composition, protein and fat (g/100g), and dry matter (g/100g) of fish species sampled from coastal Tanzania and Mozambique during the Nansen survey of 2018 and 2023. Values are presented as means ± standard deviations (SD) of the fish species analysed and expressed as the nutrient content per 100 g raw, edible part. Number of pooled samples analysed (n). Each pooled sample consisted of a minimum of 5 fish.

| **Sampled species** | **Tissue analysed** | **n** | **Protein (g/100g)** | **Fat**  **(g/100gm)** | **Dry matter (g/100g)** |
| --- | --- | --- | --- | --- | --- |
| **Tanzania** |  |  |  |  |  |
| **Small fish (< 25cm)** |  |  |  |  |  |
| *Decapterus kurroides* | W | 1 | 20 | 3.4 | 26.8 |
| *Encrasicholina heteroloba* | W | 2 | 20 ± 1 | 2.2 ± 0.1 | 24.5 ± 1.0 |
| *Spratelloides gracilis* | W | 3 | 21 ± 1 | 2.4 ± 0.5 | 25.4 ± 1.3 |
| *Upeneus taenopterus d* | W | 2 | 18 ± 0 | 4.9 ± 0.1^***^ | 27.6 ± 0.4 |
| *Encrasicholina punctifer* | W | 1 | 18 | 2.5 | 23.7 |
| *Decapterus macrosoma* | W | 1 | 20 | 4.7 | 28.2 |
| *Carangoides malabaricus* | W | 1 | 20 | 4.6 | 29.4 |
| *Amblygaster sirm^1^* | D | 1 | 20 | 1.1 | 24.81 |
| *Dussumieria acuta^2^* | D | 2 | 20 ± 0 | 1.8 ± 0.1 | 24.6 ± 0.2 |
| *Encrascicholina Intermedia^3^* | W | 3 | 20 ± 0.6 | 2.7 ± 0.5 | 25.7 ± 0.8 |
|  | H&G | 3 | 21 ± 1.2 | 1.7 ± 0.4 | 25.3 ± 0.9 |
| *Encrasicholina pseudoheteroloba^3^* | W | 3 | 21 ±1.2 | 2.7 ±0.4 | 26.7 ± 0.9 |
|  | H&G | 3 | 21 ± 0.6 | 1.9 ± 0.5 | 25.9 ± 0.8 |
| *Restrelliger krnagurta^3^* | D | 3 | 21 ± 0.6 | 1.4 ± 0.1 | 25.1 ± 0.8 |
| *Sardinella gibossa^1^* | D | 2 | 22 ± 0.7 | 3.1 ± 0.5^***^ | 28.7 ± 0.9^***^ |
| *Spratelloides gracilis^2^* | W | 2 | 21 ± 0.0 | 3.1 ± 0.0^***^ | 28.1 ± 0.6^***^ |
| *Stolephorus indicus^3^* | W | 3 | 19 ± 1.0 | 1.2 ± 0.2 | 23.7 ± 0.2 |
|  | H&G | 3 | 20 ±0.6 | 1.1 ± 0.2 | 23.2 ± 0.1 |
| **Large fish( >25 cm)** |  |  |  |  |  |
| *Trichiurus lepturus ^2^* | F | 6 | 20 ± 1.0 | 2.0 ± 0.1 | 21.5 ± 0.2 |
| *Saurida undosquamis* | F | 3 | 20 ± 1.0 | 2.6 ± 0.4 | 23.5 ± 1.1 |
| *Scomberomorus commerson* | F | 2 | 23 ± 0.0 | 0.8 ± 0.0^***^ | 25.0 ± 0.0 |
| **Mozambique** |  |  |  |  |  |
| **Small fish** |  |  |  |  |  |
| *Decapterus russelli ^2^* | W | 6 | 19 ± 0.0 | 2.3 ± 0.5 | 25.1 ± 1.3 |
|  | D | 6 | 20 ± 2.0 | 1.6 ± 0.6 | 23.1 ± 3.0 |
| *Ommastrephes bartramii ^2^* | W | 6 | 20 ± 1.1 | 2.8 ± 0.3 | 24.9 ± 1.1 |
|  | D | 6 | 18 ± 1.0 | 2.4 ± 0.4 | 22.5 ± 1.1 |
| *Upeneus japonicas* | W | 3 | 15 ± 1.0 | 2.5 ± 0.3 | 20.9 ± 1.3 |
|  | D | 3 | 18 ± 3.0 | 1.5 ± 0.1 | 20.9 ± 3.6 |
| *Upeneus taeniopterus* | W | 3 | 19 ± 1.1 | 5.3 ± 0.5^***^ | 28.3 ± 1.0 |
|  | D | 3 | 19 ± 0.0 | 3.2 ± 0.2^***^ | 22.5 ± 0.1 |
| *Decapterus macrosoma* | W | 3 | 19 ± 1.2 | 2.9 ± 0.4 | 25.3 ± 0.7 |
|  | D | 3 | 20 ± 1.0 | 1.1 ± 0.2^***^ | 23.6 ± 1.4 |
| *Saurida undosquamis ^2^* | W | 6 | 22 ± 1.4 | 1.5 ± 0.1 | 28.2 ± 3.8 |
|  | D | 6 | 24 ± 4.0 | 1.1 ± 0.2 | 28.1 ± 5.1 |
| *Engraulis capensis* | W | 3 | 19 ± 1.0 | 1.7 ± 0.1 | 24.2 ± 0.4 |
|  | D | 3 | 17 ± 1.1 | 1.1 ± 0.1 | 19.1 ± 0.5 |
| **Large fish** |  |  |  |  |  |
| *Polysteganus coeruleopunctatus* | F | 3 | 20 ± 1.0 | 1.4 ± 0.2 | 22.7 ± 0.2 |
| *Merluccius paradoxus* | F | 3 | 16 ± 0.0 | 1.5 ± 0.3 | 18.3 ± 0.8 |
| *Pomadasys kaakan* | F | 2 | 20 ± 0.0 | 0.8 ± 0.1^***^ | 20.5 ± 0.0 |
| *Scomberomorus commerson^2^* | F | 4 | 21 ± 1.0 | 1.0 ± 0.1 | 23.0 ± 1.3 |

*** p ≤ 0.0001 significant differences in fat concentration among species; ;(^1,2,3)^ indicate the number of stations where samples were collected; Abbreviations: n: number of pooled samples; Definitions: W- (Whole–head, viscera and tail included in the analysis); D- (Dressed – head, viscera and tail not included); F- (Fillets only included); H&G- (Headed and gutted-head and viscera not included)
