# Supplementary material for: Nutrient Composition of Marine Fish Species From the East African Coast: Implications for Food and Nutrition Security
Source: Food Sci Nutr. 2026 Jan 13;14(1):e71159. doi: 10.1002/fsn3.71159 (PMC12796853; doi:10.1002/fsn3.71159)
Supplement: Supplementary file 4 — Table S2: fsn371159‐sup‐0004‐TableS2.docx. [file FSN3-14-e71159-s002.docx]

**Table S2:** Fatty acid composition of fish species, tissue analysed, number of pooled samples (n) from coastal water of Tanzania during the Nansen survey of 2018 and 2023. The sum of SFA, MUFA, PUFA, n-3, n-6 and the content of EPA and DHA are presented as means ± standard deviations (SD) (g/100g) and per cent of the total of lipids in 100 g raw, edible part. Number of pooled samples analysed (n). Each pooled sample consisted of a minimum of 5 fish.

| **Sampled species** | **Tissue analysed** |  | **Sum SFA** | **Sum MUFA** | **Sum PUFA** | **Sum n-3** | **Sum n-6** | **EPA** | **DHA** |
| --- | --- | --- | --- | --- | --- | --- | --- | --- | --- |
|  |  | **n** | **g/100g (% ^e^)** | **g/100g (% ^e^)** | **g/100g (% ^e^)** | **g/100g (% ^e^)** | **g/100g (% ^e^)** | **g/100g (% ^e^)** | **g/100g (% ^e^)** |
| **Small fish (<25cm)** |  |  |  |  |  |  |  |  |  |
| *Decapterus kurroides* | W | 1 | 1.09(40) | 0.37(13) | 1.15 (42) | 0.94 (35) | 0.19 (7) | 0.19 (7) | 0.66 (24) |
| *Encrasicholina heteroloba* | W | 2 | 0.48±0.02  (38) | 0.16±0.01  (12) | 0.60±0.06  (47) | 0.49±0.05  (38) | 0.10±0.01  (8) | 0.09±0.00^abcd^  (7) | 0.36±0.04  (28) |
| *Spratelloides gracilis* | W | 3 | 0.55±0.15  (36) | 0.16±0.03  (11) | 0.76±0.21  (50) | 0.64±0.20  (42) | 0.11±0.01  (7) | 0.11±0.04  (7) | 0.48±0.12  (31) |
| *Upeneus taenopterus* | W | 2 | 1.67±0.08  (41) | 0.75±0.06  (18) | 1.58±0.07^***^  (39) | 1.18±0.02^***^  (29) | 0.38±0.08^***^  (9) | 0.32±0.03^***^  (8) | 0.70±0.07^***^  (17) |
| *Encrasicholina punctifer* | W | 1 | 0.58 (39) | 0.17 (12) | 0.66 (45) | 0.55(38) | 0.09 (7) | 0.11 (8) | 0.38 (26) |
| *Decapterus macrosoma* | W | 1 | 1.41(37) | 0.66 (18) | 1.59 (42) | 1.29 (34) | 0.28 (7) | 0.26 (8) | 0.87 (23) |
| *Carangoides malabaricus* | W |  | 1.79(42) | 0.84(19) | 1.60(37) | 1.27(30) | 0.30(7) | 0.32(7) | 0.76(18) |
| *Amblygaster sirm^1^* | D | 1 | 0.05 (34) | 0.02(11) | 0.05 (21) | 0.06 (41) | 0.01 (7) | 0.01 (4) | 0.05 (34) |
| *Dussumieria acuta^2^* | D | 2 | 0.27 ± 0.07  (42) | 0.09 ± 0.03  (14) | 0.24 ± 0.09  (38) | 0.19 ± 0.07  (30) | 0.05 ± 0.02  (7) | 0.05 ± 0.02  (7) | 0.13 ± 0.05  (21) |
| *Encrascicholina Intermedia^3^* | W | 3 | 0.63 ± 0.35  (39) | 0.20 ± 0.11  (13) | 0.65 ± 0.36  (43) | 0.54 ± 0.30  (36) | 0.10 ± 0.05  (6) | 0.11 ± 0.07  (8) | 0.36 ± 0.20  (25) |
|  | H&G | 3 | 0.25 ± 0.04  (38) | 0.08 ± 0.01  (12) | 0.29 ± 0.03  (41) | 0.24 ± 0.03  (34) | 0.04 ± 0.00  (6) | 0.05 ± 0.01  (7) | 0.16 ± 0.02  (23) |
| *Encrasicholina pseudoheteroloba^3^* | W | 3 | 0.83 ± 0.19  (40) | 0.27 ± 0.05^***^  (13) | 0.81 ± 0.08^**^  (42) | 0.66 ± 0.06^***^  (35) | 0.14 ± 0.02  (7) | 0.14 ± 0.01  (7) | 0.44 ± 0.03^***^  (24) |
|  | H&G | 3 | 0.54 ± 0.15  (38) | 0.17 ± 0.04b  (12) | 0.60 ± 0.11  (39) | 0.49 ± 0.09  (32) | 0.10 ± 0.02  (7) | 0.10 ± 0.02  (7) | 0.34 ± 0.05  (21) |
| *Restrelliger karnaguta^3^* | D | 3 | 0.20 ± 0.05  (36) | 0.06 ± 0.01  (12) | 0.24 ± 0.05  (43) | 0.18 ± 0.03  (33) | 0.06 ± 0.01  (10) | 0.02 ± 0.00  (5) | 0.14 ± 0.25  (26) |
| *Sardinella gibossa^1^* | D | 2 | 0.62 ± 0.48  (40) | 0.19 ± 0.15  (13) | 0.60 ± 0.44  (40) | 0.49 ± 0.36  (32) | 0.11 ± 0.08  (7) | 0.09 ± 0.07  (6) | 0.35 ± 0.25  (23) |
| *Spratelloides gracilis^2^* | W | 2 | 0.82 ± 0.00  (39) | 0.28 ± 0.02^***^  (14) | 0.85 ± 0.05^**^  (41) | 0.72 ± 0.04^***^  (35) | 0.12 ± 0.01  (6) | 0.15 ± 0.01  (7) | 0.50 ± 0.02^***^  (24) |
| *Stolephorus indicus^3^* | W | 3 | 0.28 ± 0.07  (38) | 0.08 ± 0.02  (11) | 0.30 ± 0.06  (47) | 0.25 ± 0.05  (40) | 0.05 ± 0.01  (7) | 0.04 ± 0.01 (  8) | 0.19 ± 0.03  (30) |
|  | H&G | 3 | 0.15 ± 0.02  (40) | 0.03 ± 0.01  (8) | 0.18 ± 0.02  (42) | 0.15 ± 0.01  (35) | 0.03 ± 0.01  (7) | 0.03 ± 0.00  (6) | 0.12 ± 0.01  (27) |
| ***Large fish(> 25cm)*** |  |  |  |  |  |  |  |  |  |
| *Trichiurus lepturus ^2^* | F | 6 | 0.44±0.05  (32) | 0.48±0.07  (35) | 0.42±0.00  (30) | 0.33±0.03  (24) | 0.09±0.01  (6) | 0.04±0.01  (3) | 0.25±0.02  (18) |
| *Saurida undosquamis* | F | 3 | 0.67±0.22  (29) | 0.80±0.21  (35) | 0.79±0.27  (0.63) | 0.63±0.22  (27) | 0.16±0.04  (7) | 0.10±0.04  (4) | 0.47±0.15  (20) |

**p ≤ 0.001 significant differences in fatty acids composition among species; *** p ≤ 0.0001 significant differences in fatty acids composition among species; ^(1,2,3)^ indicate number of stations where samples were collected. Abbreviations: n: number of pooled samples; DHA: docosahexaenoic acid; EPA: eicosapentaenoic acid; MUFA: monounsaturated fatty acids; PUFA: polyunsaturated fatty acids; SD: standard deviation, SFA: saturated fatty acids; Definitions: W- (Whole–head, viscera and tail included in the analysis); D- (Dressed – head, viscera and tail not included); F- (Fillets only included) H&G- (Headed and gutted-head and viscera not included)
