# Supplementary material for: Nutrient Composition of Marine Fish Species From the East African Coast: Implications for Food and Nutrition Security
Source: Food Sci Nutr. 2026 Jan 13;14(1):e71159. doi: 10.1002/fsn3.71159 (PMC12796853; doi:10.1002/fsn3.71159)
Supplement: Supplementary file 5 — Table S3: fsn371159‐sup‐0005‐TableS3.docx. [file FSN3-14-e71159-s007.docx]

**Table S3:** Fatty acid composition of fish species, tissue analysed, number of pooled samples (n) from coastal water of Mozambique during the Nansen survey of 2018. The sum of SFA, MUFA, PUFA, n-3, n-6 and the content of EPA and DHA are presented as means ± standard deviations (SD) (g/100g) and per cent of the total lipids in 100 g raw per 100 g raw, edible part. Number of pooled samples analysed (n). Each pooled sample consisted of a minimum of 5 fish.

| **Sampled species** | **Tissue analysed** | **n** | **Sum SFA** | **Sum MUFA** | **Sum PUFA** | **Sum n-3** | **Sum n-6** | **EPA** | **DHA** |
| --- | --- | --- | --- | --- | --- | --- | --- | --- | --- |
|  |  |  | **g/100g (% ^e^)** | **g/100g (% ^e^)** | **g/100g (% ^e^)** | **g/100g (% ^e^)** | **g/100g (% ^e^)** | **g/100g(% ^e^)** | **g/100g(% ^e^)** |
| **Small fish (<25cm)** |  |  |  |  |  |  |  |  |  |
| *Decapterus russelli ^2^* | W | 6 | 0.47±0.16  (33) | 0.18±0.06  (12) | 0.68±0.19  (49) | 0.56±0.15  (41) | 0.11±0.04  (8) | 0.11±0.04  (7) | 0.40±0.08 (29) |
|  | D | 6 | 0.44±0.24  (32) | 0.16±0.1  (11) | 0.68±0.23^d^  (53) | 0.57±0.18  (44) | 0.11±0.04  (8) | 0.09±0.04  (7) | 0.42±0.11 (33) |
| *Ommastrephes bartrandi ^2^* | W | 6 | 0.45±0.06  (33) | 0.10±0.02  (7) | 0.77±0.14  (57) | 0.67±0.13  (50) | 0.09±0.01  (7) | 0.17±0.06  (12) | 0.50±0.08 (37) |
|  | D | 6 | 0.36±0.07  (34) | 0.07±0.03  (7) | 0.60±0.13  (57) | 0.54±0.12  (51) | 0.06±0.01  (6) | 0.13±0.05 (12) | 0.40±0.08 (38) |
| *Upeneus japonicas* | W | 3 | 0.76±.02  (38) | 0.36±0.01  (18) | 0.84±0.02  (41) | 0.66±0.02  (33) | 0.17±0.01  (8) | 0.16±0.00  (8) | 0.43±0.01 (21) |
|  | D | 3 | 0.44±0.08  (38) | 0.17±0.03  (15) | 0.53±0.09  (46) | 0.42±0.07  (37) | 0.10±0.01  (9 | 0.09±0.01  (8) | 0.30±0.04 (26) |
| *Upeneus taeniopterus* | W | 3 | 1.52±0.30^***^  (41) | 0.81±0.17^***^  (22) | 1.23±0.14^***^  (34) | 0.95±0.10  (26) | 0.27±0.03^***^  (7) | 0.31±0.04  (8) | 0.52±0.05  (14) |
|  | D | 3 | 0.97±0.05  (40) | 0.49±0.04  (21) | 0.86±0.05  (36) | 0.66±0.04  (28) | 0.19±0.01  (8) | 0.20±0.02  (8) | 0.38±0.02 (16) |
| *Decapterus macrosoma* | W | 3 | 0.67±0.13  (35) | 0.24±0.06  (12) | 0.93±0.12  (48) | 0.77±0.10  (40) | 0.15±0.02  (8) | 0.16±0.03  (8) | 0.53±0.06  (27) |
|  | D | 3 | 0.37±0.05  (33) | 0.11±0.01  (10) | 0.59±0.05  (53) | 0.50±0.04b  (44) | 0.09±0.01  (8) | 0.09±0.01  (8) | 0.37±0.03  (33) |
| *Saurida undosquamis ^2^* | W | 6 | 0.28±0.04  (35) | 0.14±0.01  (17) | 0.33±0.05  (41) | 0.26±0.03  (25) | 0.07±0.01  (9) | 0.04±0.01  (5) | 0.20±0.02 (25) |
|  | D | 6 | 0.22±0.07  (33) | 0.09±0.04  (14) | 0.32±0.12  (48) | 0.27±0.11  (26) | 0.05±0.01  (8) | 0.04±0.01  (6) | 0.21±0.09 (32) |
| *Engraulis capensis* | W | 3 | 0.41±0.04  (36) | 0.11±0.01  (10) | 0.55±0.05  (49) | 0.48±0.05  (43) | 0.07±0.01  (6) | 0.09±0.01  (8) | 0.36±0.04 (32) |
|  | D | 3 | 0.18±0.0  (33) | 0.03±0.00  (6) | 0.33±0.03  (59) | 0.30±0.03  (53) | 0.03±0.00  (5) | 0.05±0.00  (9) | 0.24±0.02 (43) |
| **Large fish (>25cm)** |  |  |  |  |  |  |  |  |  |
| *Polysteganus coeruleopunctatus* | F | 3 | 0.21±0.05  (32) | 0.09±0.05  (13) | 0.33±0.05  (50) | 0.25±0.04  (39) | 0.07±0.01 (11) | 0.03±0.01  (5) | 0.20±0.03 (32) |
| *Merluccius paradoxus* | F | 3 | 0.25±0.05  (27) | 0.27±0.08  (28) | 0.39±0.05  (42) | 0.34±0.04  (37) | 0.05±0.01  (5) | 0.06±0.01  (7) | 0.25±0.02 (27) |
| *Pomadasys kaakan* | F | 2 | 0.06±0.02  (27) | 0.03±0.01  (12) | 0.14±0.05  (57) | 0.08±0.03  (35) | 0.05±0.01  (21) | 0.01±0.00  (5) | 0.06±0.02 (26) |
|  |  |  |  |  |  |  |  |  |  |
| *Scomberomorus commerson ^2^* | F | 4 | 0.21±0.06  (37) | 0.08±0.03  (13) | 0.27±0.05  (48) | 0.22±0.04  (38) | 0.05±0.01  (9) | 0.03±0.01  (5) | 0.17±0.03 (31) |
|  |  |  |  |  |  |  |  |  |  |

; *** p ≤ 0.0001 significant differences in fatty acids compositions among species; ^2^ fish sampled from two locations. Abbreviations: n: number of pooled samples; DHA: docosahexaenoic acid; EPA: eicosapentaenoic acid; MUFA: monounsaturated fatty acids; PUFA: polyunsaturated fatty acids; SD: standard deviation, SFA: saturated fatty acids; Definitions:W- (Whole–head, viscera and tail included in the analysis); D- (Dressed – head, viscera and tail not included); F- (Fillets only included); H&G- (Headed and gutted-head and viscera not included).
