# Supplementary material for: Nutrient Composition of Marine Fish Species From the East African Coast: Implications for Food and Nutrition Security
Source: Food Sci Nutr. 2026 Jan 13;14(1):e71159. doi: 10.1002/fsn3.71159 (PMC12796853; doi:10.1002/fsn3.71159)
Supplement: Supplementary file 6 — Table S4: fsn371159‐sup‐0006‐TableS4.docx. [file FSN3-14-e71159-s004.docx]

**Table S4:** Vitamin content of fish species sampled from coastal water of Tanzania and Mozambique during the Nansen survey of 2018 and 2023. Values are presented as means ± standard deviations (SD) of the fish species analysed and expressed as the nutrient content per 100 g raw, edible part. Number of pooled samples analysed (n). Each pooled sample consisted of a minimum of 5 fish.

| **Sampled species** | **Tissue analysed** | **n** | **Vitamin A1**  **(µg/100g)** | **Vitamin A2**  **(µg/100g)** | **Vitamin B9 (µg/100g )** | **Vitamin B12 (µg/100g)** |
| --- | --- | --- | --- | --- | --- | --- |
| **Tanzania** |  |  |  |  |  |  |
| **Small fish (<25cm)** |  |  |  |  |  |  |
| *Decapterus kurroides* | W | 1 | 230 | 19 | ND | 9.5 |
| *Encrasicholina heteroloba* | W | 2 | 12 0± 57^***^ | 12 ± 7.1^***^ | ND | 9.5 ± 0.6 |
| *Spratelloides gracilis* | W | 3 | 28 ± 24^***^ | 1.5 ± 0.0 | ND | 8.8 ± 0.4 |
| *Upeneus taenopterus* | W | 2 | 260 ± 28^***^ | 24 ± 7.8^***^ | ND | 9.6 ± 0.3 |
| *Encrasicholina punctifer* | W | 1 | 100 | 8 | ND | 9.4 |
| *Decapterus macrosoma* | W | 1 | 490 | 42 | ND | 8.1 |
| *Carangoides malabaricus* | W | 1 | 500 | 31 | ND | 12 |
| *Amblygaster sirm^1^* | D | 1 | 20 | 2.2 | 32 | 10 |
| *Dussumieria acuta^2^* | D | 2 | 27 ± 28 | 1.4 ± 2.0 | 15 ± 2.1^**^ | 4.5 ± 1.2 |
| *Encrascicholina Intermedia^3^* | W | 3 | 22 ± 4.2 | <LOQ | 64 ± 12 | 10.3 ± 0.6 |
|  | H&G | 3 | 3.3 ± 1.1^*^ | <LOQ | 38 ± 5.6 | 8.4 ± 0.5 |
| *Encrasicholina pseudoheteroloba^3^* | W | 3 | 44 ± 31 | 3.2 ± 2.5 | 42 ± 4.4 | 6.8 ± 2.1 |
|  | H&G | 3 | 11 ± 9.1 | 0.8 ± 1.4 | 25 ± 6.0 | 6.5 ± 1.2 |
| *Restrelliger kanagurta^3^* | D | 3 | 14 ± 8.9 | <LOQ | 31 ± 17 | 7.0 ± 1.9 |
| *Sardinella gibossa^1^* | D | 2 | 34 ± 11 | 18 ± 15 | 49 ± 4.9 | 9.2 ± 0.6 |
| *Spratelloides gracilis^2^* | W | 2 | 34 ± 2.8 | <LOQ | 33 ± 5.7 | 12.5 ± 0.7^***^ |
| *Stolephorus indicus^3^* | W | 3 | 17 ± 9.6 | 2.3 ± 1.1 | 31 ± 3.2 | 4.1 ± 0.1 |
|  | H&G | 3 | 12 ± 7.6 | 1.4 ± 0.3 | 25 ± 3.6 | 3.5 ± 0.2 |
| **Large fish (>25cm)** |  |  |  |  |  |  |
| *Trichiurus lepturus ^2^* | F | 6 | 17 ± 2.6^***^ | <LOQ | ND | 0.9 ± 0.2^***^ |
| *Saurida undosquamis* | F | 3 | 28 ± 19^***^ | 0.5±0.0^***^ | ND | 1.8 ± 0.2^***^ |
| **Mozambique** |  |  |  |  |  |  |
| **Small fish (<25cm)** |  |  |  |  |  |  |
| *Decapterus russelli ^2^* | W | 6 | 110 ± 28^***^ | 5.6 ± 1.0 | 32 ± 3.9^***^ | 7.7 ± 2.0 |
|  | D | 6 | 9.6 ± 3.0 | <LOQ | 11 ± 1.8 | 7.4 ± 1.9 |
| *Ommastrephes bartramii ^2^* | W | 6 | 22 ± 3.0 | <LOQ | 20 ± 0.5^***^ | 4.5 ± 1.2 |
|  | D | 6 | 8.7 ± 2.7 | <LOQ | 4.9 ± 1.7 | 3.0 ± 0.4 |
| *Upeneus japonicas* | W | 3 | 170 ± 30^***^ | 12 ± 2.6^***^ | 27 ± 6.2^***^ | 8.4 ± 0.8^***^ |
|  | D | 3 | 4.7 ± 3.0 | <LOQ | 7.2 ± 0.6 | 4.0 ± 1.9 |
| *Upeneus taeniopterus* | W | 3 | 120 ± 30^***^ | 6.7 ± 2.1 | 41 ± 1.2^***^ | 6.6 ± 0.6 |
|  | D | 3 | 8.3 ± 0.6 | 0.6 ± 0.1 | 8.8 ± 0.8 | 4.8 ± 0.5 |
| *Decapterus macrosoma* | W | 3 | 100 **±** 17^***^ | 8.3 ± 2.1 | 45 ± 3.1^***^ | 10 ± 0.8 |
|  | D | 3 | 12 ± 6.7 | 1.2 ± 0.0 | 20 ± 3.8 | 10 ± 1.3 |
| *Saurida undosquamis* | W | 6 | 56 ± 12 | 2.4 ± 1.9 | 36 ± 5.4^***^ | 5.7 ± 1.6*** |
|  | D | 6 | 2.6 ± 0.9^***^ | <LOQ | 13 ± 3.6 | 1.7 ± 0.3 |
| *Engraulis capensis* | W | 3 | 113 ± 40 | 8.7 ± 3.5^***^ | 33 ± 2.5^***^ | 4.2 ± 0.4 |
|  | D | 3 | 4.3 ± 1.5^***^ | 0.7 ± 0.1 | 3.7 ± 0.9 | 3.1 ± 0.1 |
| **Large fish (>25cm)** |  |  |  |  |  |  |
| *Polysteganus coeruleopunctatus* | F | 3 | 0.1 ± 0.4 | <LOQ | 5.6 ± 0.4 | 1.4 ± 0.3 |
| *Merluccius paradoxus* | F | 3 | 12 ± 7.0 | 0.8 ± 0.3^***^ | 19 ± 3.5 | 0.8 ± 0.2^***^ |
| *Pomadasys kaakan* | F | 2 | 5.0 ± 0.0 | <LOQ | 6.7 ± 1.6 | 1.1 ± 0.1 |
| *Scomberomorus commerson ^2^* | F | 4 | 5.0 ± 3.6 | 0.8 ± 0.1*** | 8.0 ± 2.1 | 1.6 ± 0.2 |

**p ≤ 0.01 significant differences in vitamin concentration among species; *** p ≤ 0.0001 significant differences in vitamin concentrations among species; ^(1,2,3)^ indicate number of stations where samples were collected. Abbreviations: n: number of pooled samples ND: not determined, LOQ: Limit of Quantification, LOQ; Vitamin A2: 0.5 µg/100 g raw, edible part; Definitions: W- (Whole–head, viscera and tail included in the analysis); D- (Dressed – head, viscera and tail not included); F- (Fillets only included); H&G- (Headed and gutted-head and viscera not included).
