# Supplementary material for: Nutrient Composition of Marine Fish Species From the East African Coast: Implications for Food and Nutrition Security
Source: Food Sci Nutr. 2026 Jan 13;14(1):e71159. doi: 10.1002/fsn3.71159 (PMC12796853; doi:10.1002/fsn3.71159)
Supplement: Supplementary file 7 — Table S5: fsn371159‐sup‐0007‐TableS5.docx. [file FSN3-14-e71159-s001.docx]

**Table S5:** Mineral composition of fish species sampled from coastal water of Tanzania and Mozambique during the Nansen survey of 2018 and 2019. Values are presented as means ± standard deviations (SD) of the fish species analysed and expressed as the nutrient content per 100 g raw, edible part. Number of pooled samples analysed (n). Each pooled sample consisted of a minimum of 5 fish.

| **Sampled species** | **Tissue analysed** | **N** | **Ca** | **Fe** | **I** | **Zn** |
| --- | --- | --- | --- | --- | --- | --- |
|  |  |  | **(mg/100g)** | **(mg/100g)** | **(µg/100g)** | **(mg/100g)** |
| **Tanzania** |  |  |  |  |  |  |
| **Small fish (<25cm)** |  |  |  |  |  |  |
| *Decapterus kurroides* | W | 1 | 850 | 2.4 | 52 | 1.4 |
| *Encrasicholina heteroloba* | W | 2 | 835 ± 106 | 1.5 ± 0.4 | 8.5 ± 2.1 | 2.9 ± 0.4 |
| *Spratelloides gracilis* | W | 3 | 875 ± 106 | 4.5 ± 3.0 | 235 ± 134^***^ | 3.0 ± 0.0 |
| *Upeneus taenopterus* | W | 2 | 1550 ± 212 | 3.1 ± 0.2 | 145 ± 21 | 1.2 ± 0.0 |
| *Encrasicholina punctifer* | W | 1 | 490 | 1.3 | 200 | 1.6 |
| *Decapterus macrosoma* | W | 1 | 850 | 4.9 | 200 | 1.9 |
| *Carangoides malabaricus* | W | 1 | 1600 | 0.2 | 85 | 1.4 |
| *Amblygaster sirm^1^* | D | 1 | 1100 | 2.2 | 53 | 2.8 |
| *Dussumieria acuta^2^* | D | 2 | 750 ± 156 | 0.9 ± 0.2 | 26 ± 12 | 2.1 ± 0.2 |
| *Encrascicholina Intermedia^3^* | W | 3 | 563 ± 116 | 1.1 ± 0.2 | 168 ± 94*** | 2.1 ± 0.2 |
|  | H&G | 3 | 353 ± 32 | 0.7 ± 0.2 | 43 ± 10 | 1.6 ± 0.1 |
| *Encrasicholina pseudoheteroloba^3^* | W | 3 | 793 ± 31 | 1.4 ± 0.1*** | 127 ± 29*** | 3.0 ± 0.4 |
|  | H&G | 3 | 440 ± 79 | 0.8 ± 0.1 | 58 ± 13 | 2.4 ± 0.4 |
| *Restrelliger krnagurta^3^* | D | 3 | 960 ± 40 | 1.4 ± 0.3 | 41 ± 17 | 1.6 ± 0.0 |
| *Sardinella gibossa^1^* | D | 2 | 1005 ± 134 | 2.3 ± 0.1*** | 103 ± 81 | 2.5 ± 0.1*** |
| *Spratelloides gracilis^2^* | W | 2 | 715 ± 205b | 2.5 ± 0.0*** | 365 ± 134*** | 2.9 ± 0.4*** |
| *Stolephorus indicus^3^* | W | 3 | 817 ± 140 | 1.0 ± 0.1 | 66 ± 18 | 2.3 ± 0.1*** |
|  | H&G | 3 | 613 ± 230 | 0.6 ± 0.1 | 39 ± 10 | 1.4 ± 0.1 |
| **Large fish (>25cm)** |  |  |  |  |  |  |
| *Trichiurus lepturus ^2^* | F | 6 | 39 ± 3^***^ | 0.2 ± 0.0 | 13 ± 1.5 | 0.3 ± 0.0 |
| *Saurida undosquamis* | F | 3 | 64 ± 15^***^ | 0.2 ± 0.0 | 14 ± 1.3 | 0.3 ± 0.0 |
| *Scomberomorus commerson* | F | 2 | 25 ± 8^**^ | 0.3 ± 0.0 | 36 ± 2.8 | 0.3 ± 0.0 |
| **Mozambique** |  |  |  |  |  |  |
| **Small fish** |  |  |  |  |  |  |
| *Decapterus russelli ^2^* | W | 6 | 968 ± 89 | 3.3 ± 2.0 | 197 ± 32^***^ | 1.4 ± 0.1 |
|  | D | 6 | 305 ± 54^***^ | 0.9 ± 0.1^***^ | 77 ± 30 | 1.0 ± 0.1^***^ |
| *Ommastrephes bartramii ^2^* | W | 6 | 32 ± 3 | 0.3 ± 0.1 | 33 ± 12 | 1.4 ± 0.2 |
|  | D | 6 | 15 ± 3^***^ | 0.1 ± 0.0 | 19 ± 2.4 | 1.1 ± 0.1^***^ |
| *Upeneus japonicas* | W | 3 | 1003 ± 167 | 2.0 ± 0.5 | 65 ± 10 | 0.9 ± 0.1 |
|  | D | 3 | 373 ± 112^***^ | 0.8 ± 0.3^***^ | 23 ± 3.6 | 0.5 ±0.1^***^ |
| *Upeneus taeniopterus* | W | 3 | 1367 ± 306 | 5.0 ± 0.3 | 130 ± 10^***^ | 1.2 ± 0.1 |
|  | D | 3 | 157 ± 47^***^ | 1.2 ± 0.4^***^ | 56 ± 6.4 | 0.4 ± 0.0^***^ |
| *Decapterus macrosoma* | W | 3 | 773 ± 12 | 2.4 ± 0.1 | 140 ± 0.0 | 1.3 ± 0.1^***^ |
|  | D | 3 | 190 ± 79^***^ | 1.0 ± 0.2 | 82 ± 13 | 0.9 ± 0.1 |
| *Saurida undosquamis ^2^* | W | 6 | 1117 ± 75 | 1.6 ± 1.0 | 38 ± 16 | 1.5 ± 0.1 |
|  | D | 6 | 310 ± 125^***^ | 1.6 ± .7 | 18 ± 7.2 | 0.6 ± 0.0^***^ |
| *Engraulis capensis* | W | 3 | 413 ± 55 | 1.4 ± 0.3 | 36 ± 0.0 | 1.8 ± 0.0 |
|  | D | 3 | 102 ± 8^***^ | 0.2 ± 0.0 | 14 ± 2.1 | 0.4 ± 0.0^***^ |
| **Large fish (>25cm)** |  |  |  |  |  |  |
| *Polysteganus coeruleopunctatus* | F | 3 | 67 ± 37^***^ | 0.2 ± 0.0 | 16.3 ± 1.2 | 0.3 ± 0.0 |
| *Merluccius paradoxus* | F | 3 | 15 ± 4^***^ | 0.1 ± 0.0 | 7.2 ± 0.8 | 0.3 ± 0.0 |
| *Pomadasys kaakan* | F | 2 | 20 ± 13^***^ | 0.3 ± 0.0 | 43 ± 9.9 | 0.3 ± 0.0 |
| *Scomberomorus commerson^2^* | F | 4 | 6 ± 1^***^ | 0.2 ± 0.0 | 17 ± 4.8 | 0.3 ± 0.0 |

**p ≤ 0.01 significant differences in mineral content among species; *** p ≤ 0.0001 significant differences in mineral concentrations among species; (^1,2,3)^ indicate the number of stations where samples were collected. analysis Abbreviations: n: number of pooled samples; Ca: Calcium, Fe: iron, I: iodine, Zn: Zinc; Definitions: W- (Whole–head, viscera and tail included in the); D- (Dressed – head, viscera and tail not included); F- (Fillets only included); H&G- (Headed and gutted-head and viscera not included).
